# Identification of two types of GGAA-microsatellites and their roles in EWS/FLI binding and gene regulation in Ewing sarcoma

**DOI:** 10.1371/journal.pone.0186275

**Published:** 2017-11-01

**Authors:** Kirsten M. Johnson, Cenny Taslim, Ranajeet S. Saund, Stephen L. Lessnick

**Affiliations:** 1 The Medical Scientist Training Program and the Biomedical Sciences Graduate Program, The Ohio State University College of Medicine, Columbus, Ohio, United States of America; 2 Center for Childhood Cancer and Blood Diseases, Nationwide Children’s Hospital Research Institute, Columbus, Ohio, United States of America; 3 Division of Pediatric Hematology/Oncology/BMT, The Ohio State University College of Medicine, Columbus, Ohio, United States of America; Tulane University School of Medicine, UNITED STATES

## Abstract

Ewing sarcoma is a bone malignancy of children and young adults, frequently harboring the EWS/FLI chromosomal translocation. The resulting fusion protein is an aberrant transcription factor that uses highly repetitive GGAA-containing elements (microsatellites) to activate and repress thousands of target genes mediating oncogenesis. However, the mechanisms of EWS/FLI interaction with microsatellites and regulation of target gene expression is not clearly understood. Here, we profile genome-wide protein binding and gene expression. Using a combination of unbiased genome-wide computational and experimental analysis, we define GGAA-microsatellites in a Ewing sarcoma context. We identify two distinct classes of GGAA-microsatellites and demonstrate that EWS/FLI responsiveness is dependent on microsatellite length. At close range “promoter-like” microsatellites, EWS/FLI binding and subsequent target gene activation is highly dependent on number of GGAA-motifs. “Enhancer-like” microsatellites demonstrate length-dependent EWS/FLI binding, but minimal correlation for activated and none for repressed targets. Our data suggest EWS/FLI binds to “promoter-like” and “enhancer-like” microsatellites to mediate activation and repression of target genes through different regulatory mechanisms. Such characterization contributes valuable insight to EWS/FLI transcription factor biology and clarifies the role of GGAA-microsatellites on a global genomic scale. This may provide unique perspective on the role of non-coding DNA in cancer susceptibility and therapeutic development.

## Introduction

Ewing sarcoma is the second most common pediatric bone malignancy, initiated by a chromosomal translocation t(11;22)(q24;q12), creating the fusion protein and oncogenic driver EWS/FLI. As an aberrant transcription factor, EWS/FLI plays a critical role in regulating genes involved in tumorigenesis [1]. Typically, FLI and other ETS family members bind DNA via their conserved DNA binding domain at the consensus sequence ‘ACCGGAAGTG’ [2,3]. This high affinity DNA binding site containing a single GGAA core motif is necessary for oncogenesis [4–6], with FLI and EWS/FLI displaying similar DNA binding affinity and specificity [7]. In Ewing sarcoma, however, EWS/FLI displays a “gain-of-function” in its ability to also bind ‘GGAA’-containing microsatellite (repeat) regions to regulate some of its targets, such as key oncogenic target *NR0B1* [8,9].

Microsatellites are tandem, or sequentially repeated DNA motifs, frequently found in or near gene promoters [10,11]. In Ewing sarcoma, repetitive “microsatellite” regions comprised of the motif “GGAA” have been identified as highly enriched EWS/FLI-bound sequences near transcription start sites of EWS/FLI up-, but not down-regulated genes [9,12]. We and others confirmed that these putative binding sites specifically confer EWS/FLI-mediated activation of their adjacent target [9,12–14]. Additionally, we recently demonstrated a relationship between the number of repeats in these regions and their ability to function as EWS/FLI-response elements: an 18–26 GGAA-motif “sweet-spot” repeat length provides maximal transcriptional function, and is significantly enriched in patients with Ewing sarcoma [15]. How polymorphisms of GGAA-microsatellites in Ewing sarcoma affect EWS/FLI binding and transcriptional regulation across the genome, however, remains unclear.

Although these GGAA-containing regions fall under the traditional definition of “microsatellites,” this term has been loosely applied in a Ewing sarcoma context to include a wide-range of “GGAA” sequences and is somewhat arbitrary, especially given their polymorphic nature [16]. Clearly defining GGAA-microsatellites in a Ewing sarcoma relevant context is needed to understand their mechanistic role in EWS/FLI transcription factor regulation. Additionally, delineating a clear relationship between microsatellite length, location and transcriptional regulation across the genome is essential. Together, these disparities represent a significant void in our understanding of EWS/FLI transcriptional biology, and remain a powerful barrier to potential therapeutic amelioration. Our previous demonstration of GGAA-microsatellites as EWS/FLI response elements, coupled with *in vitro* and clinical data indicating a “sweet-spot” length, suggest a relationship between EWS/FLI and these unique binding sites in transcriptional activation [9,16]. Here, we sought to define GGAA-microsatellites in a Ewing sarcoma context, and to understand their role across the genome.

To accomplish this, we use bioinformatics analysis of experimental data to first characterize GGAA-microsatellites, setting pre-determined parameters for an unbiased genome-wide approach. Once described, we then computationally link bound microsatellites to adjacent EWS/FLI regulated genes. Our data reveal two distinct types of GGAA-microsatellites: close-range (“promoter-like”) and long-range (“enhancer-like”), and suggest differing mechanisms of EWS/FLI-mediated activation and repression at these elements. Classification of these clarifies the genome-wide presence of GGAA-microsatellites in Ewing sarcoma and their role in transcriptional regulation.

## Materials and methods

### Cell culture

The Ewing sarcoma cell line A673 from ATCC was cultured, and retroviruses packaged in HEK293-EBNA cells, using standard procedures described previously [17,18]. For RNA interference experiments, cells were infected with pMSCV-puro retrovirus harboring shRNA constructs against luciferase (control) or EWS/FLI.

### Searching for GGAA repeat regions

Human reference genome (hg19) was scanned to find the occurrences of GGAA and TTCC using Biostrings [19] and BSgenome [20] R packages. An in-house script was used to find a region that contains multiple GGAA-motifs not separated by more than 20 non-GGAA nucleotides. The region has to start and end with GGAA. The same procedure was used to find repeat regions with TTCC-motifs. Each region was then annotated with its nearest gene (pseudo genes were filtered from annotation database) using ChIPpeakAnno [21] R package.

### ChIP-seq analysis

Chromatin immunoprecipitation (ChIP) was performed as previously described [22] using anti-FLI-1 (Santa Cruz, sc-356X Santa Cruz Biotechnology, Inc.). Briefly, chromatin from formaldehyde-fixed A673 cells was fragmented to a size range of 200–700 bases with a Misonix Sonicator. Solubilized chromatin was immunoprecipitated with anti-FLI-1 and antibody-chromatin complexes were pulled down with M-280 sheep anti-rabbit IgG Dynabeads (Thermo Scientific), washed and then eluted. After crosslink reversal, RNAse A and Proteinase K treatment, immunoprecipitated DNA was extracted with the Mini-Elute PCR purification kit (Qiagen). ChIP DNA was quantified with Qubit, libraries prepared and sequenced with Illumina HiSeq 2500. Raw sequence reads can be found in NCBI’s Gene Expression Omnibus database under GSE99959. Sequence reads were aligned to the human reference genome (hg19) using Novoalign (http://novocraft.com). Duplicate reads were removed using samtools [23]. Peaks were identified using MACS2 [24] at FDR cut-off of 5%. To assess whether GGAA-repeat regions overlap with EWS/FLI binding sites more than one would expect by chance, we used permutation tests implemented in regioneR R library [25]. Overlap is defined as region with ≥ 1 bp overlap. Specifically, we compared the number of overlap in the actual EWS/FLI binding sites and GGAA-repeat regions (with at least 3 consecutive repeats) to that seen in a random sample of universe regions (i.e. resampleRegions strategy in regioneR library). Since GGAA-repeat regions with at least three consecutive motifs are a subset of all repeat regions in the genome, we used all repeat regions as the universe regions. This randomization strategy maintained the internal structure of GGAA-repeats. A different randomization strategy (i.e. randomizeRegions) which randomly places repeat regions along the mappable regions of the genome was also performed with similar results (data not shown). Although significant, the association between EWS/FLI binding sites and GGAA-repeat regions with at least three consecutive motifs might be indirect and based on the fact that both regions tend to cluster around gene-rich regions. In order to check whether this association is specifically linked to the relative position of these two regions with each other, we shifted the regions and evaluated the z-score for every shifted position. The sharp peak, as shown in S1C Fig, indicates this association is highly dependent on the relative position of the two regions with each other, and the association is not regional. In order to do a correlation test, we associated each microsatellite with its nearest EWS/FLI peak binding sites (distance is calculated from the middle of the microsatellite to EWS/FLI peak summit location). Correlation coefficients (*r*) were calculated using Spearman’s correlation.

### RNA-seq analysis

The RNA-seq data set used in this work was previously published [26]. Briefly, RNA collected from A673 cells stably infected and selected for expression of a control Luc-RNAi or the EF-2-RNAi was extracted using the RNAeasy kit (Qiagen) with an on-column DNAse digestion protocol. Libraries for deep-sequencing were prepared according to the manufacturer’s instructions (Illumina) and sequenced on an Illumina Hi-Seq 2000 with 50-bp single end reads. Sequences were aligned to the human genome build hg19 using Novoalign (http://novocraft.com). Raw sequence reads can be found in the NCBI SRA under SRA059239. Gene model used for counting reads/fragments were from Ensembl GRCh37 (release 75) GTF [27]. R packages GenomicAlignments [28], GenomicFeatures [28] and BiocParallel [29] were used to count the number of reads/fragments assigned to genomic features in each sample. Genomic features with total counts less than 2 across samples were removed. Data quality was assessed by clustering all samples. Normalized rlog (regularized log transformation) counts [30] and pheatmap [31] R package were used to do hierarchical clustering. S11 Fig shows a heatmap of sample-to-sample distance. Differential gene analysis was done using DESeq2, which uses negative binomial modeling and the empirical Bayes shrinkage method for fold-change estimation [30].

### Correlations between EWS/FLI binding intensities, EWS/FLI-regulated gene expressions and microsatellites

All **c**orrelations were calculated using Spearman’s rank correlation. LOESS regression (Local Polynomial regression fitting) line and its t-based approximation of 95% confidence bands were drawn using R library ggplot2 [32]. We used Loess regression because of its advantage as robust to outliers and its ability to show non-linear association [33].

### Data availability

RNA-seq raw sequence reads can be found in the NCBI SRA under SRA059239. ChIP-seq raw sequence reads can be found in NCBI’s Gene Expression Omnibus database under GSE99959

## Results

### GGAA-motifs in a microsatellite occur on the same strand

**Fig 1:**
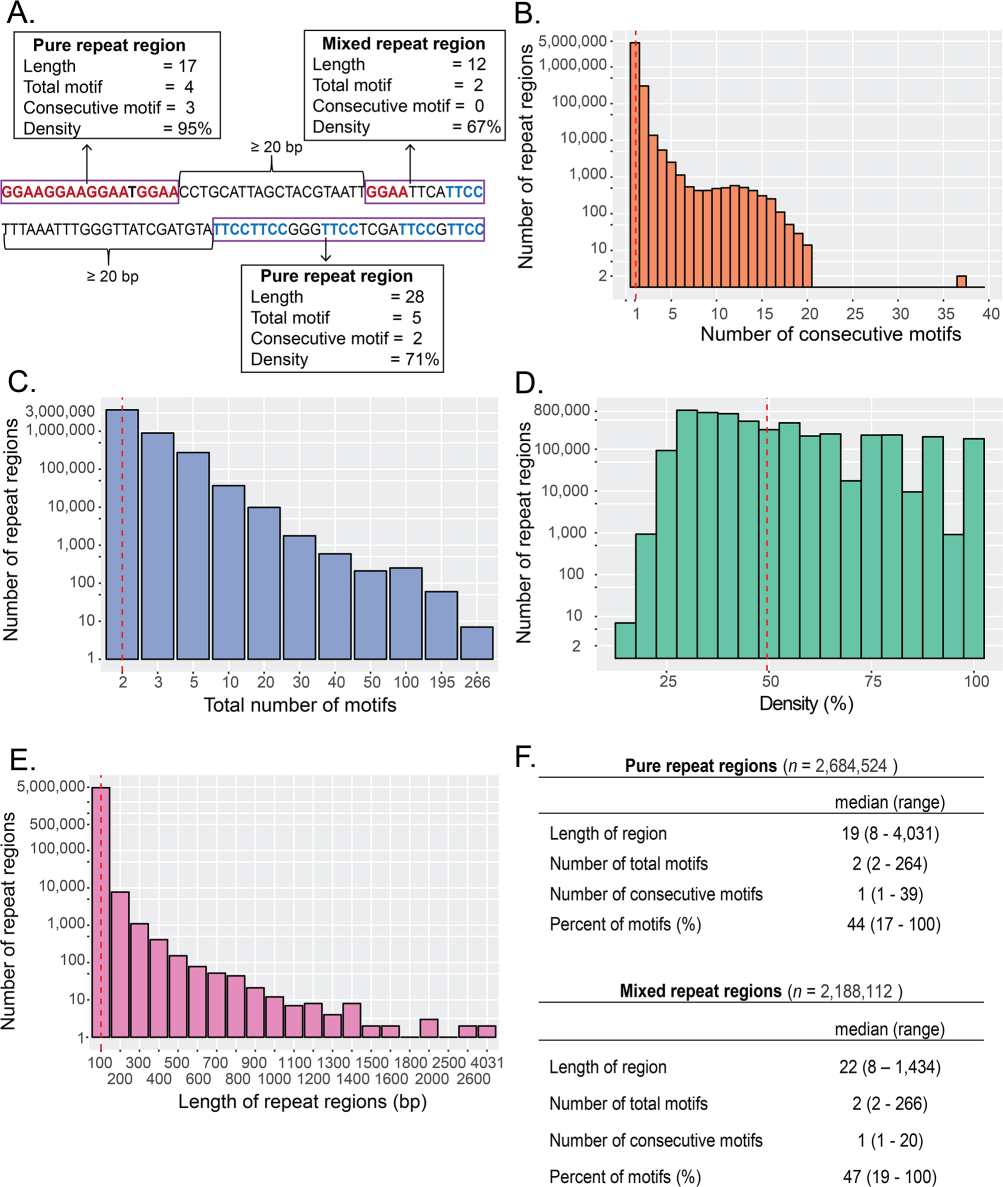
Schema and characteristics of repeat regions across genome. (**A**) Schema of repeat regions. Regions with only one type of motif are called pure repeat region while those with both GGAA and TTCC are called mixed repeat regions. Each repeat region (purple box) is separated by at least 20-bp consecutive non-motifs. (**B**) Histogram of maximum number of consecutive motifs. (**C**) Histogram of total number of motifs. (**D**) Histogram of motif density of repeat regions. $Density=\left(\frac{total\;number\;of\;motifs\times 4}{length\;of\;regions}\right)\times 100\%$. Bin width is 5%. (**E**) Histogram of length of repeat regions. Each bin is 100bp width (e.g., first bin is 0-100bp length). Bins with zero repeat regions are not shown. (**F**) The characteristics of repeat regions for pure and mixed repeat regions across the genome. Red line indicates the mean for each characteristic.

EWS/FLI, the aberrant transcription factor in Ewing sarcoma, modulates gene expression by binding to GGAA-containing repetitive regions [9]. However, genome-wide characterization of these repeat regions is lacking, including whether microsatellites with GGAA-motifs are present on both strands of DNA. We first scanned the human reference genome (hg19) on both strands for GGAA-motifs. We defined a repeat region as a sequence that starts and ends with a GGAA-motif and which has no more than 20 insertions (non-motif nucleotides) between two adjacent motifs (Fig 1A). Nearly 5 million repeat regions span the genome. Although the total number of motifs in any given region ranges from 2 to 266 motifs, 3.7 million regions contain less than 3 motifs (Fig 1B and 1C). These sparse repeat regions have an average GGAA content, or density, of around 50% (Fig 1D and 1E). Additionally, most of these repeat regions have no consecutive motifs (93.2%) and less than 0.6% has at least 3 consecutive motifs.

Given typical FLI binding within the major groove of DNA at GGAA-containing regions [34], we considered the possibility of GGAA-motifs existing on both strands of DNA within the same repeat region. Multiple EWS/FLI molecules could conceivably bind adjacent motifs on alternating strands of DNA and thereby avoid steric hindrance in binding [6]. We classified repeat regions that contain GGAA-motifs on the same strand as pure repeat regions, while regions that include GGAA on both forward and reverse strands are referred to as mixed repeat regions (Fig 1A). We determined more than half of the 5 million GGAA-repeat regions across the genome (55%) contain GGAA-motifs on the same strand (Fig 1F).

To determine whether a microsatellite can contain mixed motifs, we looked specifically at mixed repeat regions with at least 3 or more consecutive motifs. We found more than 81% of them contain only a single GGAA-motif on the opposite strand. While only 32 regions (1.2% of 2,589) have 2 or more consecutive GGAA-motifs on both strands in the same region, even in these rare examples motifs cluster together on the same strand. Additionally, only one region has more than 2 consecutive GGAA-motifs on both strands (S1 Table). Based on these observations, we deduced *bona fide* microsatellites with GGAA-motifs on both strands may not exist in the same region. This finding prompted us to re-process mixed repeat regions, separating clusters of GGAA-motifs as two distinct regions if they are on opposite strands. Thus we discounted repeat regions with only one GGAA-motif on each strand, leaving 3,321,889 repeat regions. We focus our downstream analysis solely on these homogenous (i.e. same strand) repeat regions. For ease of reading, henceforth we will refer to these GGAA-motifs simply as repeat regions.

### Longer GGAA-regions are located near genes while shorter GGAA-regions are ubiquitous across the genome

**Fig 2:**
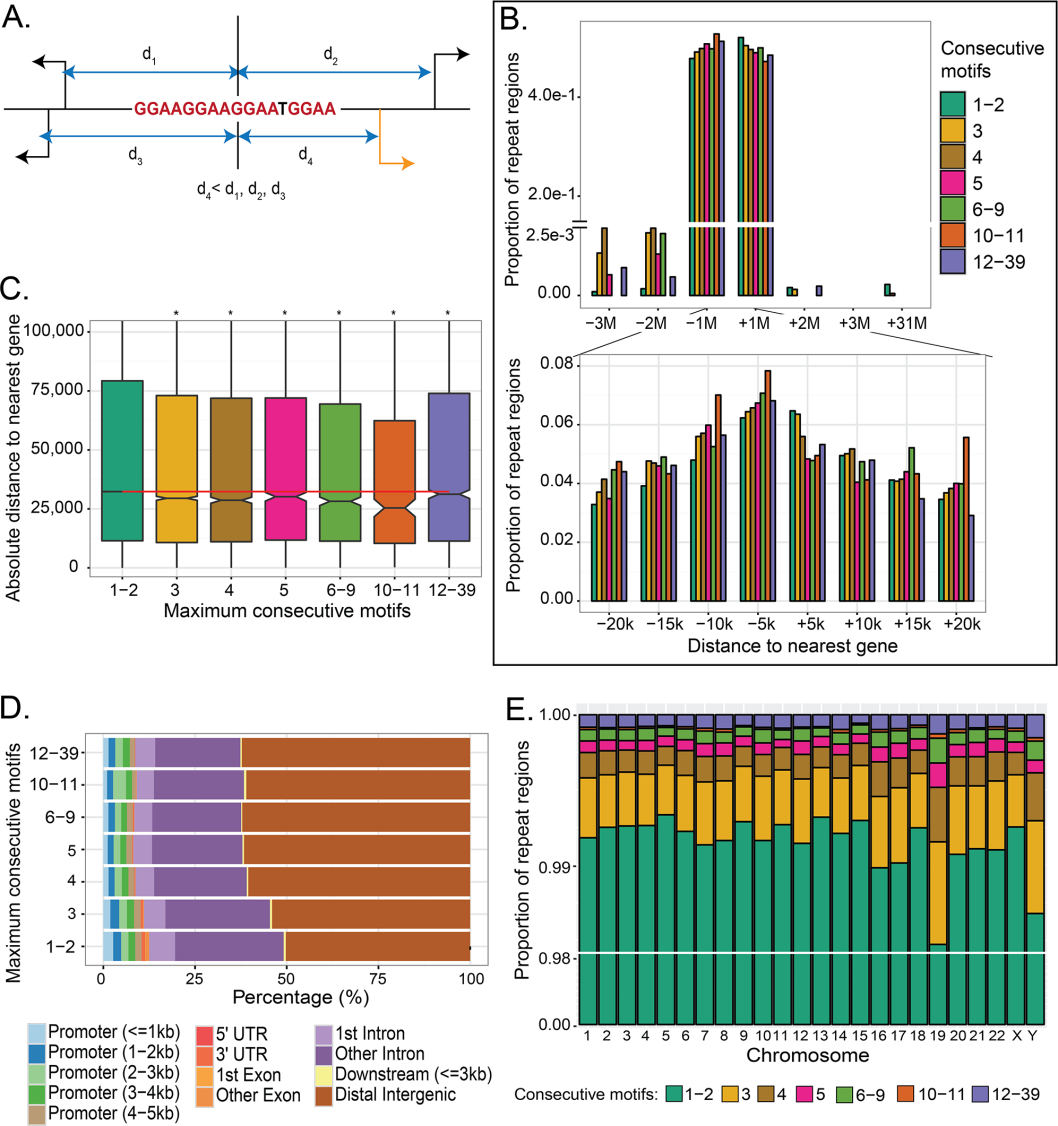
Nearest gene schema and genomic location of repeat regions. (**A**) Schema showing the nearest gene (orange) which is the gene with the shortest distance calculated from its TSS to the middle of the repeat region. (**B**) Distribution of distances to nearest genes for each repeat region grouped by number of consecutive motifs. The sum of percentages for each consecutive motif is 100%. (**C**) Comparisons of distance-to-nearest-gene for longer consecutive motifs to repeat regions with one to two consecutive motifs (i.e. ‘1–2’). * indicates the repeat regions are significantly closer to a gene than repeat regions with 1–2 consecutive motifs (*p* < 0.05). Red line represents the median distance-to-nearest gene for repeat regions with 1–2 consecutive motifs. (**D**) Feature distribution for each consecutive motif category. (**E**) Proportions of repeat regions in each chromosome grouped by the number of consecutive motifs.

Of the more than 3 million repeat regions in the genome, we found 99% of them contain only two consecutive motifs. In many of these regions, these motifs likely happen by chance and consequently have no function. A subset of these regions, however, may act as EWS/FLI response elements, driving regulation of critical oncogenic gene targets such as *NR0B1* [9]. To facilitate functional analysis of these repeat regions in an unbiased approach, we started by annotating each repeat region with its nearest genes and observing the distribution of these repeat regions in terms of both their nearest genes and genomic location. The nearest gene is the gene with the shortest distance from the center of the GGAA-microsatellite to the transcription start site (TSS), regardless of strand direction (Fig 2A). Most GGAA-regions occur within 3Mb of a gene. Notable exceptions include 1,355 regions with 1 or 2 consecutive motifs and a single 3-consecutive motif region that are greater than 30Mb away from a gene (Fig 2B).

Although many repeat regions with two or less consecutive motifs reside near the TSS, on average most are farther away from genes compared to regions with longer consecutive motifs (t-tests, *p* < 0.05) (Fig 2C). Conversely, we found that repeat regions with 10–11 consecutive motifs are closer on average to genes than other consecutive motifs and are slightly enriched in promoter regions of genes 2 to 3kb from the TSS (Fig 2C and 2D). This enrichment of repeat regions with 10–11 consecutive motifs near genes suggests possible preferential binding of EWS/FLI at these microsatellites.

Looking by individual chromosome, we demonstrated that repeat regions with 1–2 consecutive motifs account for 99% of GGAA-containing regions (Fig 2E). Interestingly, chromosome 19 has a higher proportion of longer consecutive motifs (more than 3 consecutive motifs) than the other chromosomes. We later found chromosome 19 also has a greater number of EWS/FLI peaks (see later discussion on EWS/FLI binding).

Overall, our data indicate that short consecutive repeat regions (less than 3 consecutive motifs) may not have any EWS/FLI related function as they are ubiquitously scattered throughout the genome. We therefore investigated whether a GGAA-microsatellite needs to have a minimum number of motifs to allow EWS/FLI binding in a Ewing sarcoma context.

### EWS/FLI bound GGAA-microsatellites contain three or more GGAA-repeats

Our previous in-vitro data indicated a minimum of three consecutive GGAA-motifs is required for EWS/FLI binding [35]. To test this requirement computationally across the genome, we addressed the following question: Does significant overlap exist between repeat regions with certain lengths and EWS/FLI binding sites? We investigated these relationships using ChIP-seq experiments in the A673 Ewing sarcoma cell line. Four paired-end ChIP-seq samples immunoprecipitated with a FLI-specific antibody were analyzed using Model Based Analysis for ChIP-seq (MACS2) [36]. 22,744 EWS/FLI binding sites were identified at a False Discovery Rate (FDR) cut-off of 0.05. Chromosome 19, which has more repeat regions at three or more consecutive motifs, also has an increased number of EWS/FLI binding sites per Mb compared to the other chromosomes (S1A Fig). This further supports defining repeats of 3 or more consecutive motifs as EWS/FLI response elements. The total repeat regions that overlap with EWS/FLI binding sites are 26,922 (Table 1).

**Table 1:** Number of repeat regions and EWS/FLI binding sites.

	EWS/FLI		no EWS/FLI		Total
GGAA-motifs	n	%	n	%	
All Repeat Regions	26,922	0.81%	3,294,967	99.19%	3,321,889
1 motif	15,615	0.51%	3,023,699	99.49%	3,039,314
2 consecutive motifs	3,051	1.19%	252,809	98.81%	255,860
3 consecutive motifs	1,570	12.14%	11,359	87.86%	12,929
4 consecutive motifs	1,536	28.29%	3,894	71.71%	5,430
5 consecutive motifs	978	38.76%	1,545	61.24%	2,523
≥ 6 consecutive motifs	4,172	71.52%	1,661	28.48%	5,833

Number of GGAA-repeat regions by number of consecutive GGAA-motif and EWS/FLI binding sites across the genome.

**Fig 3:**
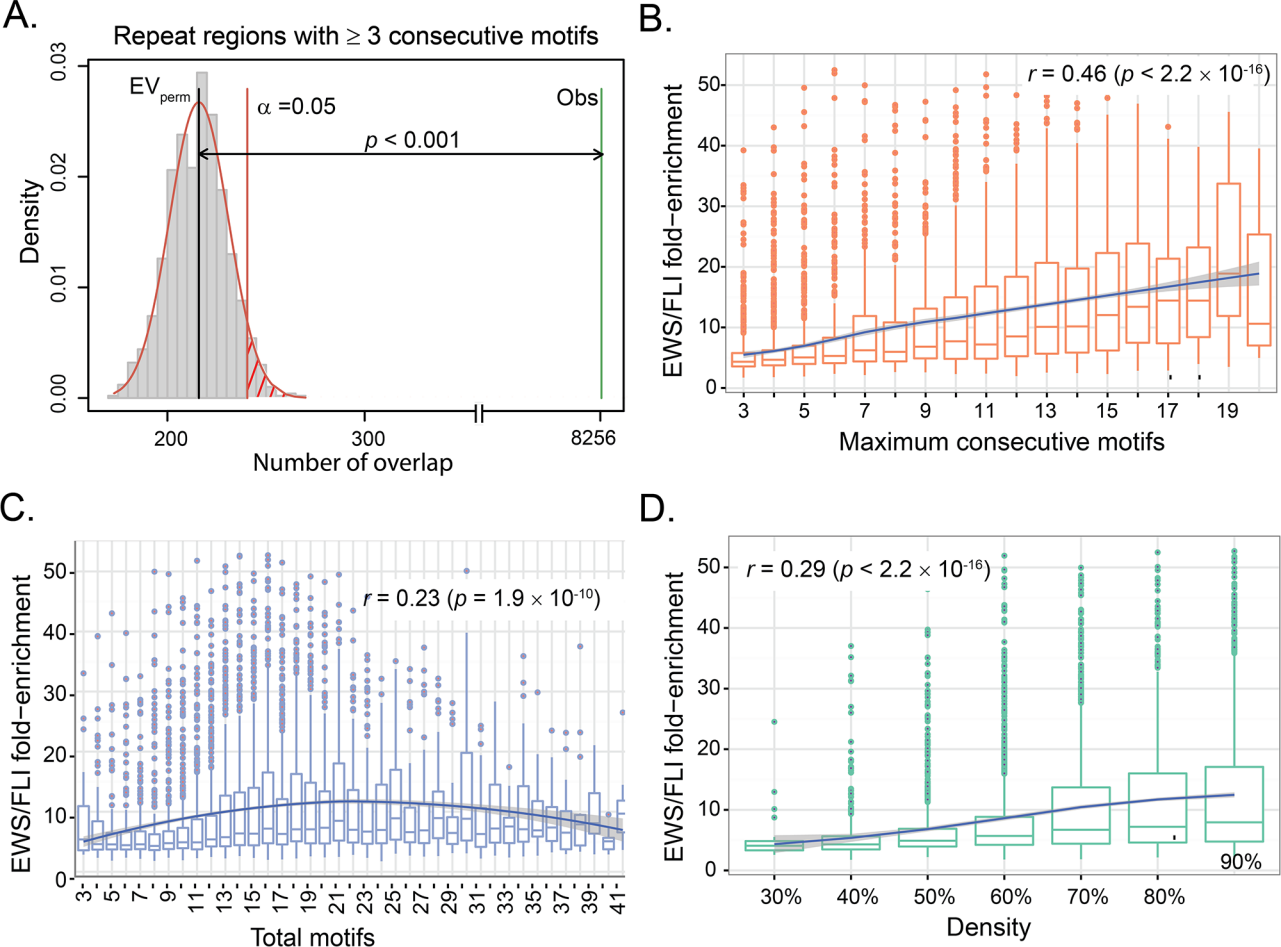
Characteristics of EWS/FLI-bound microsatellites. (**A**) Permutation test shows that the number of EWS/FLI binding sites that overlap with repeat regions (*n* = 8,256) with minimum of 3 consecutive motifs is significantly higher than random chance (*p* < 0.001). Red line denotes the significance limit (α = 0.05). Gray bars represent the number of overlaps in the random regions with EWS/FLI binding sites in 1,000 permutations. The black line represents the mean of overlaps in random regions (EV_perm_) and the green bar is the actual number of overlaps observed in repeat regions (Obs). (**B**) Boxplot of EWS/FLI fold-enrichment (relative to genomic background) and number of consecutive motifs in EWS/FLI-bound microsatellites showing statistically significant increasing trend (*p* < 2.2 × 10^−16^). The blue line is the estimated LOESS regression line of the mean with the estimated 95% confidence bands (shaded region). (**C**) Boxplot of EWS/FLI fold-enrichment and total number of motifs in EWS/FLI-bound microsatellites showing a positive correlation (*p* = 1.9 × 10^−10^) and a non-linear trend (*p* < 0.05). The blue line is the estimated LOESS regression line of the mean with the estimated 95% confidence bands (shaded region). (**D**) Boxplot of EWS/FLI fold-enrichment and Density $\left(=\frac{total\;motif\times 4}{length\;of\;microsatellite}\times 100\%\right)$ showing statistically significant positive correlation (*p* < 2.2 × 10^−16^). The blue line is the estimated LOESS regression line of the mean with the estimated 95% confidence bands (shaded region).

To evaluate whether the amount of overlap between repeat regions and EWS/FLI binding sites occurs by chance, we assessed statistical significance with a permutation test. We observed repeat regions with three or more consecutive motifs overlap significantly with EWS/FLI binding sites (*p* < 0.001, Fig 3A), while repeat regions with two or less consecutive motifs do not overlap significantly (*p* = 1, S1B Fig). This finding is consistent with our experimental observation that a minimum of three consecutive GGAA-motifs is required for EWS/FLI binding [35].When we randomly move the locations of repeat regions with longer consecutive motifs across the genome, we observe a sharp decrease in the statistical significance of overlap with EWS/FLI binding sites. This decrease in overlap indicates that the association is not regional but is highly dependent on motif location (S1C Fig). Based on the combination of these observations, we now define GGAA-microsatellites as repeat regions with 3 or more consecutive motifs. Downstream analyses focus on GGAA-microsatellites according to this definition.

### Increasing number of GGAA-motifs correlates with increased EWS/FLI binding intensity

Having defined GGAA-microsatellites, we next investigated EWS/FLI binding at these regions to determine whether GGAA-motif enrichment in a given microsatellite region affects binding of EWS/FLI at that genomic loci. We defined the closest microsatellite to the center of the EWS/FLI binding site as the putative EWS/FLI-bound microsatellite. We grouped the 6,031 EWS/FLI-bound microsatellites (S2 Table) by number of consecutive motifs (S1D Fig). Since we found only 11 EWS/FLI-bound microsatellites with more than 20 consecutive motifs, statistical evaluation and inclusion of these data points were difficult and uninformative. We therefore excluded microsatellites with more than 20 consecutive motifs in this analysis.

Overall, we demonstrate a positive correlation between EWS/FLI binding intensity and the number of consecutive motifs contained by these EWS/FLI-bound microsatellites (*r* = 0.46, *p* < 2.2×10^−16^) (Fig 3B and S2 Fig). This genome-wide trend of overall increasing EWS/FLI binding enrichment with increasing number of consecutive motifs is consistent with our previous *in-vitro* study [37].

Most EWS/FLI-bound microsatellites have 11 to 19 total motifs with a maximum of 195 motifs (S3 Fig). We see a similar positive correlation between EWS/FLI binding and total motifs (*r* = 0.23, *p* = 1.9 × 10^−10^) as with consecutive motifs (Fig 3C). We also observe a non-linear relationship between EWS/FLI fold-enrichment and total motifs, with the EWS/FLI fold-enrichment increasing from 3 to about 16 total motifs, then decreasing again around 24–25 total motifs (LOESS regression) (Fig 3C and S4 Fig). These data are in agreement with our recent finding that 18–26 motifs are the optimal length for EWS/FLI binding [16]. To see whether overall GGAA content within a microsatellite affects EWS/FLI binding, we then evaluated the relationship between GGAA-motif density within a microsatellite and EWS/FLI binding enrichment. We found that EWS/FLI fold-enrichment demonstrates a statistically significant positive correlation with GGAA-motif density (*r* = 0.29, *p* < 2.2×10^−16^) (Fig 3D). These EWS/FLI-bound microsatellite densities range from 30% to 90%, with most EWS/FLI-bound microsatellites (>1,500) having a density of 90% (S5 Fig).

Overall, our analysis shows a positive correlation between number of motifs and overall GGAA content, which increases with increased EWS/FLI binding. There is also a non-linear trend between EWS/FLI fold-enrichment and total motifs, implicating an optimal, or “sweet-spot”, microsatellite length for EWS/FLI binding similar to our recent study [38].

### EWS/FLI gene regulation at associated GGAA-microsatellites

In the previous section, we established the global correlation between microsatellite motif number and EWS/FLI binding intensities. Though this correlation allowed us to define GGAA-microsatellites in terms of length based on bound EWS/FLI, transcription factor binding is not always indicative of transcriptional regulation [39]. To determine whether GGAA-microsatellite characteristics are predictive of EWS/FLI responsiveness at a given genomic loci, we evaluated both the expression of EWS/FLI target genes and binding intensity associated with these microsatellites. We and others previously showed that EWS/FLI regulates its activated, but not repressed targets through binding at GGAA-microsatellites [35]. Prior analysis of these regions, however, has primarily focused on microsatellites located within about 5kb of associated EWS/FLI target promoters [9]. We therefore separately evaluated EWS/FLI activated and repressed targets associated with microsatellites both near (within 5kb) and distal (greater than 5kb) to the TSS of these genes. Differential gene expression profiles grouped based on these distinct categories of activated vs. repressed and close-range vs. distal microsatellites were integrated and stratified by distance of each GGAA-microsatellite to the nearest gene. Gene expression profiles were derived from six independent RNA-seq experiments on wild type vs. EWS/FLI knock-down A673 cells. DESeq2 [30] identified 9,323 differentially expressed (4,278 activated and 5,045 repressed) genes between control and treatment cell lines at a FDR of 5%.

#### EWS/FLI binding and gene activation at promoter-like microsatellites is highly dependent on the length of GGAA-motifs

**Fig 4:**
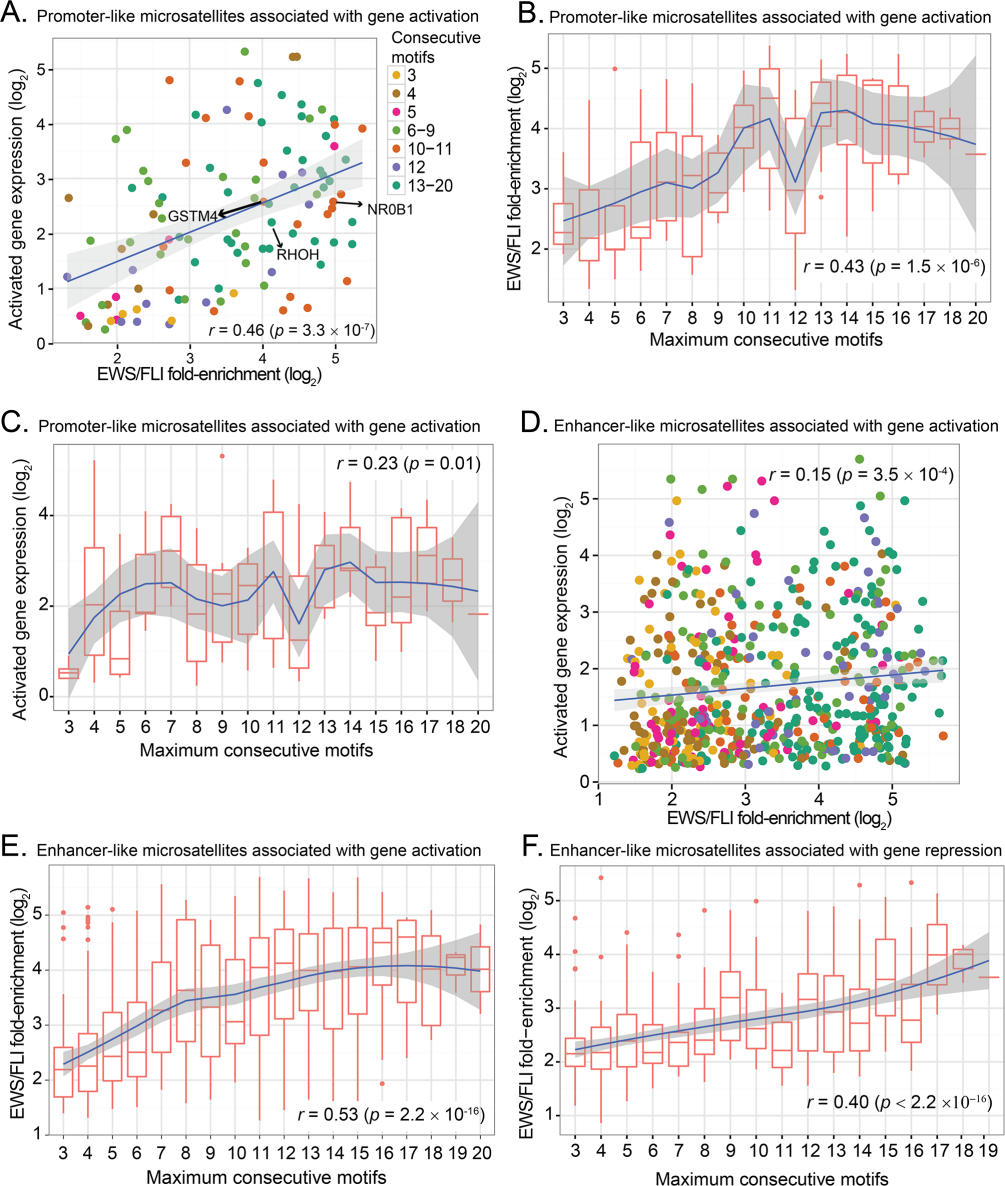
Correlation between EWS/FLI-bound microsatellites, GGAA-motif and gene expression. (**A**) Scatter plot of expression of activated genes and EWS/FLI fold-enrichment at promoter-like microsatellites showing a positive correlation (*r* = 0.46, *p* = 3.35 × 10^−7^). (**B**) Boxplot of EWS/FLI fold-enrichment and number of consecutive motifs of EWS/FLI-bound at promoter-like microsatellites for activated genes showing a non-linear trend. Blue line is the estimated LOESS regression line of the mean with the estimated 95% confidence interval (shaded region). Overall, there is statistically significant positive correlation (*r* = 0.43, *p* = 1.5 × 10^−6^). (**C**) Boxplot of EWS/FLI-activated gene expression and number of consecutive motifs at promoter-like EWS/FLI-bound microsatellites for gene activation showing a non-linear trend as seen in EWS/FLI binding intensities and a statistically significant positive correlation (*r* = 0.23, *p* = 0.01). The blue line is the estimated LOESS regression line of the mean with the estimated 95% confidence bands (shaded region). (**D**) Scatter plot of expression of activated genes and EWS/FLI fold-enrichment at enhancer-like microsatellites showing a positive correlation (*r* = 0.15, *p* = 3.5 × 10^−4^). (**E**) Boxplot of EWS/FLI fold-enrichment and number of consecutive motifs at EWS/FLI-bound enhancer-like microsatellites showing a positive correlation (*r* = 0.53, *p* = 2.2 × 10^−16^). Blue line is the estimated LOESS regression line of the mean and the standard error of the prediction shown as shaded region. (**F**) Boxplot of EWS/FLI fold-enrichment and number of consecutive motifs at EWS/FLI-bound enhancer-like microsatellites associated with gene repression showing positive correlation (*r* = 0.40, *p* < 2.2 × 10^−16^). The blue line is the estimated LOESS regression line of the mean with the estimated 95% confidence bands (shaded region).

There are 114 microsatellites within 5kb of activated genes. To see if EWS/FLI binding at these close-range, promoter-like, microsatellites confer gene activation, we looked at EWS/FLI binding enrichment and gene expression for these microsatellites. As anticipated based on our previous studies, we found increased EWS/FLI binding correlates with expression of activated target genes (Fig 4A and S6 Fig) (*r* = 0.46, *p* = 3.3×10^−7^). Furthermore, increasing number of consecutive GGAA-motifs correlates with increased EWS/FLI binding intensity (*r* = 0.43, *p* = 1.5×10^−6^) and also increases in subsequent gene activation (*r* = 0.23, *p* = 0.01) (Fig 4B and 4C). EWS/FLI binding and total GGAA-motif number and density, demonstrate a trend toward positive correlation, though not significant for these microsatellites (S7A–S7C Fig).

We also observe, however, a non-linear pattern, with an increasing trend of EWS/FLI binding as consecutive motifs increase from 3 to 11, followed by a sharp decrease in binding at 12 consecutive motifs (Fig 4B). Binding then increases again at 13–14 consecutive motifs before a final overall decreasing trend (LOESS regression). Interestingly, we observe a similar non-linear pattern with the expression of genes activated by EWS/FLI (i.e. an increase in the activated gene expressions as the consecutive motifs increases from 3 to 11 and a decrease in gene expression from 11–12) (Fig 4C, LOESS regression).

We next sought to validate these findings using publically-available data from a different Ewing sarcoma cell line, SK-N-MC. The publically-available SK-N-MC data contained a single ChIP-seq replicate and had significantly fewer EWS/FLI-bound peaks than we found in the A673 cell line (~3,900 versus ~22,000) [13]. Nevertheless, we found a low, but statistically significant correlation between consecutive GGAA-microsatellite length and EWS/FLI binding (S7D Fig). Interestingly, we saw the same dip in EWS/FLI binding at 12-repeats as we observed in the A673 data, perhaps indicating an underlying biological mechanism worthy of future study. We also sought to correlate gene expression with microsatellite length and EWS/FLI occupancy; however, an insufficient number of genes passed the significance threshold used for our A673 data and so these correlations could not be performed. Overall, there is a positive correlation between EWS/FLI binding, activated gene expression, and microsatellite characteristics (i.e. consecutive motifs, total motifs and densities), though the correlation of microsatellite characteristics with EWS/FLI binding enrichment is consistently stronger than with gene expression (S2 Table). These observations demonstrate that as promoter-like microsatellite length (number of GGAA-motifs) increases, the EWS/FLI binding enrichment and expression of genes activated by EWS/FLI also increases. This finding also supports the “sweet-spot” model, suggesting there may be an optimal length of promoter-like microsatellites mediating EWS/FLI regulation of transcriptional gene activation.

#### At enhancer-like microsatellites, increased numbers of GGAA-motifs positively correlate with EWS/FLI binding but only minimally with gene activation

To determine whether longer-range, enhancer-like microsatellites also confer EWS/FLI activation associated with motif length, we next looked at the 580 microsatellites that are more than 5kb away from EWS/FLI activated genes. We observe a minimal (significant) positive linear correlation between EWS/FLI binding enrichment and gene expression for these long-range potential response elements (*r* = 0.15, *p* = 3.5×10^−4^) (Fig 4D). Evaluating total number of motifs in these microsatellites, we observe a significant positive correlation with EWS/FLI binding enrichment (*r* = 0.25, *p* = 1.28×10^−9^), and a minimal positive correlation with EWS/FLI activated gene expression (*r* = 0.10, *p* = 0.02) (See S8A and S8B Fig and S2 Table). We also observe a significant positive linear correlation between EWS/FLI enrichment and the number of consecutive motifs of these microsatellites (*r* = 0.53, *p* < 2.2×10^−16^) (Fig 4E), and a minimal, non-significant positive trend with EWS/FLI activated gene expression (*r* = 0.07, *p* = 0.08) (S8C Fig). These observations suggest that although longer GGAA-motifs enhance EWS/FLI binding, it only minimally translates to an increase in the expression of activated gene targets. This is likely due to the complexity of long-range regulatory mechanisms.

#### At promoter-like microsatellites, number of GGAA-motifs demonstrates no length-dependency with EWS/FLI responsiveness for gene repression

To test whether GGAA-microsatellite characteristics affect EWS/FLI-mediated repression, we first looked at the 52 promoter-like microsatellites that are within 5kb of EWS/FLI-repressed genes. In contrast to EWS/FLI-activated genes, there is no significant correlation between microsatellite characteristics (i.e. number of consecutive motifs and total number of motifs) and EWS/FLI binding enrichment or EWS/FLI-mediated gene repression. The correlation between EWS/FLI binding enrichment and EWS/FLI-regulated genes is 0.12 (*p* = 0.41) (S9A Fig). Correlation of EWS/FLI binding enrichment with number of consecutive motifs is 0.18 (*p* = 0.19) and with total number of motifs is 0.06 (*p* = 0.66) (S9B and S9C Fig). We also observed no correlation between EWS/FLI-repressed genes with the number of consecutive motifs (*r* = -0.22, *p* = 0.12) and total number of motifs (*r* = -0.04, *p* = 0.79) (See S3 Table and S9D and S9E Fig). Overall, promoter-like GGAA-microsatellites don’t enhance either EWS/FLI binding or expression of repressed genes, supporting the model that EWS/FLI represses gene targets through an alternate regulatory mechanism.

#### At enhancer-like microsatellites, number of GGAA-motifs positively correlates with EWS/FLI binding but not gene repression

To test whether enhancer-like microsatellites confer EWS/FLI-mediated repression, we investigated EWS/FLI responsiveness at the 425 microsatellites that are more than 5kb away from EWS/FLI-repressed genes. Our data demonstrates increasing number of consecutive motifs positively correlates with EWS/FLI binding enrichment (*r* = 0.40, *p* < 2.2×10^−16^) (Fig 4F). Increased EWS/FLI binding enrichment is also shown to be positively correlated with total number of motifs (*r* = 0.22, *p* = 3.0×10^−6^) at these microsatellites (S10A Fig). We found, however, there is no significant correlation between EWS/FLI binding and expression of repressed genes more than 5kb from their associated microsatellite (*r* = -0.05, *p* = 0.33) (S10B Fig). Accordingly, there is also no correlation between gene expression and number of consecutive motifs or total number of motifs (*p* = 0.43 and *p* = 0.68) for these EWS/FLI-repressed gene associated microsatellites (S3 Table and S10C Fig). In summary, EWS/FLI binding increases with increasing GGAA-motif length at long-range, enhancer-like microsatellites, however, there is no effect on concomitant gene repression of these EWS/FLI targets.

## Discussion

**Fig 5:**
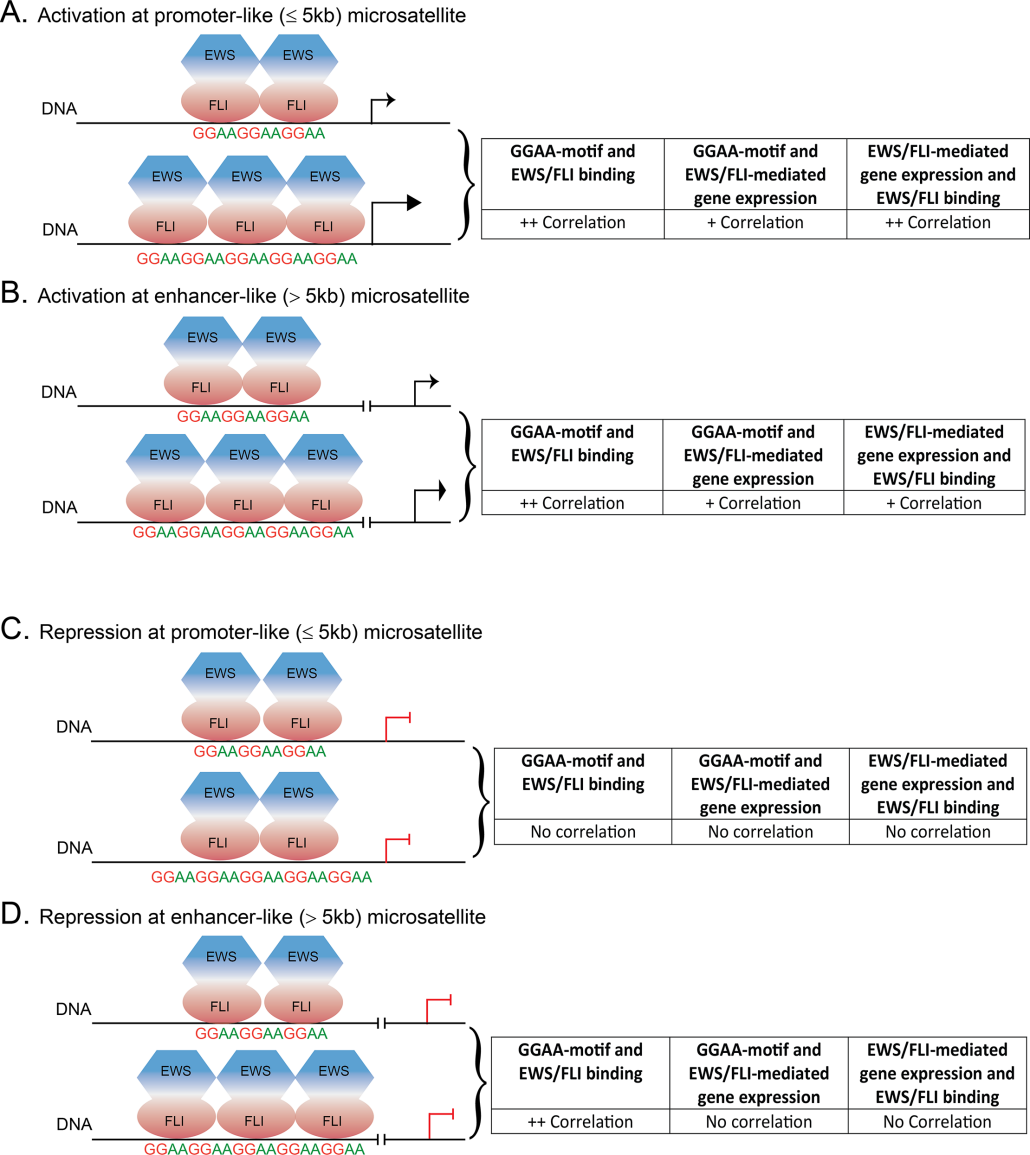
Schema of correlative associations between GGAA motifs in EWS/FLI-bound microsatellites for gene activation and repression. Schematic illustrating EWS/FLI responsiveness at given loci across the genome. (**A**) Promoter-like (close-range) GGAA-microsatellites positively correlate with EWS/FLI binding and activation of genes in a length dependent manner. (**B**) Enhancer-like (long-range) GGAA-microsatellites positively correlate with EWS/FLI binding but correlation with transcriptional regulation is only minimal for activated genes. (**C**) Promoter-like GGAA-microsatellites display no correlation with EWS/FLI binding and transcriptional repression. (**D**) Enhancer-like GGAA-microsatellites positively correlate with EWS/FLI binding; however, they do not confer gene expression.

Gene-associated GGAA-microsatellites serve as DNA response elements for EWS/FLI to bind and mediate transcriptional activation of its up-regulated targets [12,16,35,37]. In this study we describe microsatellites on a global genomic scale, and use ChIP-seq and RNA-seq analysis to computationally investigate EWS/FLI responsiveness at these repetitive elements. Overall, our genome-wide characterization of GGAA-microsatellites identifies two distinct classes of EWS/FLI-bound GGAA-microsatellites, demonstrating the integral relationship of microsatellite length and gene proximity to facilitate EWS/FLI binding and transcriptional activity in Ewing sarcoma (Fig 5).

While we and others have previously described these GGAA-microsatellites, we recognized a paucity of definitive parameterization required for mechanistic understanding of EWS/FLI transcriptional modulation at these response elements. Pursuing an unbiased genome-wide approach, we found microsatellites of fewer than three consecutive GGAA-motifs do not significantly overlap with EWS/FLI binding sites, suggesting a minimum length of consecutive motifs is required for binding. This was in line with our previous experimental finding of multimeric EWS/FLI binding at a minimum of three consecutive GGAA-repeats [35]. Thus, our genome-wide description of GGAA-microsatellite regions in this study lays an unprejudiced groundwork upon which actual ChIP-seq and RNA-seq data can be overlain. For example, in describing genome-wide GGAA-microsatellite regions, we found an enrichment of longer consecutive GGAA-repeats on chromosome 19. When FLI-ChIP-seq data was applied to the analysis, we found a corresponding enrichment of EWS/FLI binding sites on the same chromosome. In this work we present, to our knowledge, the first attempt to determinately define GGAA-microsatellites across the genome in a Ewing sarcoma-relevant context.

We and others previously showed that EWS/FLI regulates its activated, but not down-regulated targets using GGAA-microsatellites as response elements [35]. The present genome-wide analysis provides further support for length-dependency of EWS/FLI responsiveness near activated, promoter-like and enhancer-like microsatellites. Interestingly, we observe a significant correlation of GGAA-repeats associated with repressed targets and binding enrichment of EWS/FLI at enhancer-like microsatellites, illuminating a novel class of microsatellites with a potentially distinct function.

Transcriptional activation and repression are both critical for EWS/FLI-mediated oncogenic function, yet, the mechanism by which EWS/FLI differentiates these functions remains unknown. The association of EWS/FLI-bound microsatellites with only activated genes supports a likely molecular mechanistic difference in transcriptional modulation of EWS/FLI up vs. down-regulated targets. This model is further supported by our recent data that members of the chromatin remodeling NuRD complex interact with EWS/FLI near its repressed, but not activated targets [40].

Our findings in this study suggest the additional possibility that distance and overall chromatin landscape may be contributing factors in transcription factor activating vs. repressive functions. For example, recent studies have demonstrated evidence for super-enhancers, which function in long-range regulation and are associated with an enrichment of activating histone marks [41,42]. EWS/FLI binding in Ewing sarcoma cells has been shown to be bound in these super-enhancer regions [13,14,43]. To our knowledge, it has not yet been evaluated whether repressive regulatory domains exist on a similar genomic scale. The data from the present study suggests GGAA-microsatellites found in promoter-like regions convey EWS/FLI-mediated gene activation, while those found in enhancer-like regions likely require more complex regulatory factors such as chromatin remodeling complexes to establish long-range interactions. Specifically, in association with gene repression, EWS/FLI may displace endogenous transcription factors disrupting enhancer activity, a mechanism proposed by Riggi et al. [13], or these regions may naturally be more nucleosome-depleted to allow EWS/FLI binding.

An additional explanation for the mechanism by which EWS/FLI modulates activation or repression of its targets could be sequence specificity upon binding to length-dependent microsatellites [44]. We recently conducted a biochemical study to investigate the molecular reasoning behind EWS/FLI binding at “sweet-spot” microsatellites. We found that EWS/FLI binding affinity improves at “sweet-spot” microsatellites, and unexpectedly requires the EWS portion of the fusion to bind these optimal numbers of GGAA-motifs[38]. Our stoichiometric data further supports a model in which multiple EWS/FLI molecules bind across these GGAA-microsatellites. The “sweet-spot” finding evidenced in both our clinical and biochemical data implicate 18–26 GGAA repeats (“sweet-spot”) as the length of GGAA-microsatellites that allow an optimal configuration of EWS/FLI binding at these sites. Our current study suggests EWS/FLI-responsive GGAA-microsatellites are enriched near activated, but not repressed EWS/FLI targets. Taken together, it is likely that our “sweet-spot” observation is due to the aforementioned biochemical mechanism, and that this multimeric EWS/FLI binding at repeat regions may facilitate EWS/FLI differentiation between activation and repression of its targets.

FLI, which contains the DNA-binding domain through which EWS/FLI directly associates with the DNA, is an ETS family member. ETS factor binding studies have demonstrated that small differences in transcription factor binding specificity contribute significantly to site selectivity [45]. While our “sweet-spot” finding supports this model of transcription factor binding site selectivity, it is not known whether total microsatellite number (microsatellite “length”), or number of *consecutive* GGAA-motifs confers this specificity. *Guillon et al*. determined that EWS/FLI shows a binding preference for 9 or more contiguous GGAA repeats, and postulated that binding at greater than 9 repeats is required for EWS/FLI-mediated activation of its up-regulated targets [12]. This is interesting in light of our present data demonstrating a peak in EWS/FLI DNA-binding and gene activation at 10–11 and 13–14 consecutive GGAA-motifs. Although minimal, due to few microsatellites longer than 20 consecutive repeats across the genome, our overall data nevertheless suggests that microsatellites with numbers of GGAA-motifs greater than the “sweet-spot” are not associated with EWS/FLI-mediated differential gene expression. Further investigation will be required to also determine the role of consecutive motif number in relation to our “sweet-spot” finding. For example, EWS/FLI regulates *NR0B1* through a “sweet-spot” microsatellite of 24 *total* motifs in the A673 cell line, but this microsatellite region contains 11 *consecutive* motifs as its longest contiguous segment. As cited in the above results, we found this same repeat length enriched near genes compared to other consecutive motifs lengths in our genome-wide microsatellite characterization.

Our study should be considered in light of some limitations that may potentially mask the magnitude of EWS/FLI association with particular microsatellite characteristics. The first relates to the use of the human reference (hg19) genome instead of the A673 Ewing sarcoma genome as a reference. To evaluate the appropriateness of using the human reference genome, we selected a number of our favorite EWS/FLI activated genes, amplified the associated GGAA-microsatellites, and sequenced these regions. We found that some are very similar to the human reference genome (i.e. *NR0B1 and FIBCD1*), while others demonstrate significant alterations in GGAA-motif number (i.e. *PINK1*) (Johnson and Taslim, unpublished observation). Interestingly, *FCGRT* is a highly up-regulated EWS/FLI target, yet contains 12 consecutive motifs according to the human reference genome. This motif length was observed as the unexpected dip in our microsatellite-defining analysis for both EWS/FLI binding and gene-expression. Sequencing the *FCGRT* microsatellite from A673 genomic DNA, however, revealed an *FCGRT*-associated microsatellite that is actually 9-consecutive GGAA-motifs in length. Together, these findings give us confidence that our data reflects the appropriate general trends in EWS/FLI responsiveness at microsatellites, but suggests a more accurate correlation will require A673 whole genome sequencing for reference. This concept may also be applied for consideration more broadly in other fields where the human reference genome has been used instead of relevant disease genomes.

Overall, our results reveal and characterize two classes of GGAA-microsatellites, suggesting EWS/FLI interacts with these unique binding sites via distinct regulatory mechanisms for distance-dependent activation and repression of its gene targets. Defining and characterizing GGAA-microsatellites is critical for understanding and prediction of EWS/FLI responsiveness across the genome. We also demonstrate the value of synergizing experimental and computational evaluation to better delineate the underlying molecular mechanisms of EWS/FLI transcription factor function and oncogenic re-programming.

## Supporting information

S1 Fig Characterization of GGAA-repeat regions across the genome. (**A**) Histogram of number of EWS/FLI peaks per Mb (normalized by chromosome length) in each chromosome. (**B**) Permutation test shows that the number of EWS/FLI binding sites that overlap with repeat regions with 2 or less consecutive motifs is not significantly higher than random chance (*p* = 1). The red line denotes the significance limit (α = 0.05). Gray bars represent the number of overlaps of the random regions with EWS/FLI binding sites. The black line represents the mean and in green the number of overlaps of repeat regions with 2 or less consecutive motifs. EVperm is the expected value of the permutation (number of overlaps in random samples). Obs is the observed number of overlap. (**C**) Plot of shifted z-score for the association between EWS/FLI repeat regions ≥ 3 consecutive motifs and EWS/FLI binding sites showing that this association is highly dependent on the location of the regions. (**D**) Number of EWS/FLI-bound microsatellites and the number of consecutive motifs in these microsatellites.
(PDF)

S2 Fig Boxplot showing the number of consecutive motifs of all EWS/FLI-bound microsatellites with the EWS/FLI fold-enrichment. The blue line is the estimated LOESS regression line of the mean with the estimated 95% confidence bands (shaded region).
(PDF)

S3 Fig Histogram showing the number of EWS/FLI-bound microsatellites grouped by the total number of motifs. (PDF)

S4 Fig Boxplot showing the total motifs of all EWS/FLI-bound microsatellites with the EWS/FLI fold-enrichment. The blue line is the estimated LOESS regression line of the mean with the estimated 95% confidence bands (shaded region).
(PDF)

S5 Fig Histogram showing number of EWS/FLI-bound microsatellites with their densities. (PDF)

S6 Fig EWS/FLI responsiveness at promoter-like microsatellites near activated gene targets. Scatter plot showing activated gene names (False Discovery Rate (FDR) ≤ 5%) that are within 5kb of microsatellites with their EWS/FLI fold-enrichment and their corresponding gene expression (log_2_). Note: some gene names are adjusted for readability.
(PDF)

S7 Fig Promoter-like microsatellites association with gene activation. (**A**) Trend toward positive correlation between total motifs of EWS/FLI-bound microsatellites and EWS/FLI fold-enrichment (log_2_). (**B**) Trend toward positive correlation between densities of EWS/FLI-bound microsatellites and EWS/FLI fold-enrichment (log_2_). (**C**) No significant correlation between total motifs of EWS/FLI bound microsatellites and activated gene expression (log_2_). LOESS regression line is shown in blue. Shaded region is the estimated 95% confidence bands.(**D**) Trend toward positive correlation between EWS/FLI fold-enrichment (log_2_) and number of consecutive motifs in SK-N-MC cells (r = 0.06, p = 0.02).
(PDF)

S8 Fig Enhancer-like microsatellites association with EWS/FLI activated genes. (**A**) EWS/FLI fold-enrichment has a significant positive correlation with total number of motifs (*r* = 0.25, *p* = 1.28 × 10^−9^). **(B)** Gene expression has significant but minimal positive correlation with total number of motifs (*r* = 0.10, *p* = 0.02). **(C)** Trend toward minimal positive correlation between activated gene expression and number of consecutive motifs (*r* = 0.07, *p* = 0.08).
(PDF)

S9 Fig Promoter-like microsatellites association with gene repression. (**A**) No correlation between EWS/FLI fold-enrichment and gene expression (*r* = 0.12, *p* = 0.41). (**B**) No correlation between EWS/FLI fold-enrichment and number of consecutive motifs (*r* = 0.18, *p* = 0.19). (**C**) No correlation between EWS/FLI fold-enrichment and total motifs (*r* = 0.06, *p* = 0.66). (**D**) No correlation between gene expression and number of consecutive motifs (*r* = -0.22, *p* = 0.12). (**E**) No correlation between gene expression and total number of motifs (*r* = -0.04, *p* = 0.79). Shaded region is the 95% confidence interval.
(PDF)

S10 Fig Enhancer-like microsatellites associated with gene repression. (**A**) Significant positive correlation between EWS/FLI fold-enrichment and number of consecutive motifs (*r* = 0.40, *p* < 2.2×10^−4^). (**B**) No correlation between EWS/FLI fold-enrichment and gene expression (*r* = -0.05, *p* = 0.33). (**C**) No correlation between repressed genes’ expression and number of consecutive motifs (*r* = -0.04, *p* = 0.43). LOESS regression line is shown in blue. Shaded region is the estimated 95% confidence bands.
(PDF)

S11 Fig RNA-seq normalization and samples similarities. (**A**) Comparison of two different normalization methods. Left panel, counts normalized by sequencing depth. Right panel, counts normalized by rlog transformation. Sequencing depth normalization still showing bias toward highly expressed genes (i.e. high variance for low expressed genes), while rlog transformation no longer shows such bias (i.e. variances are stabilized across genes). (**B**) Heat map of sample-to-sample similarities using rlog transformed counts. Color represents distance between samples with dark blue indicating samples with high similarities.
(PDF)

S1 Table Examples of mixed repeat regions (repeat regions that contain both GGAA and TTCC motifs). (PDF)

S2 Table Correlation between microsatellites and EWS/FLI binding enrichment and EWS/FLI-activated genes. (PDF)

S3 Table Correlation between microsatellites, EWS/FLI binding enrichment and EWS/FLI-repressed genes. (PDF)
