# Supplementary figures and images for: Identification of two types of GGAA-microsatellites and their roles in EWS/FLI binding and gene regulation in Ewing sarcoma
Source: PLoS One. 2017 Nov 1;12(11):e0186275. doi: 10.1371/journal.pone.0186275 (PMC5665490; doi:10.1371/journal.pone.0186275)

S1 Fig

A.

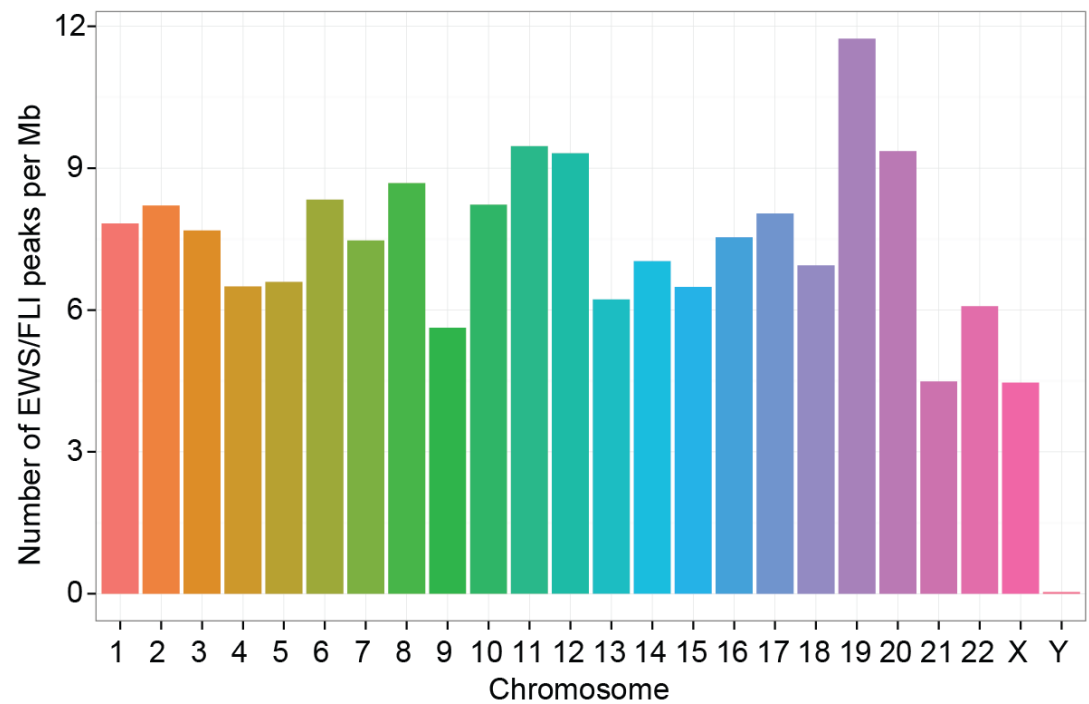

B.

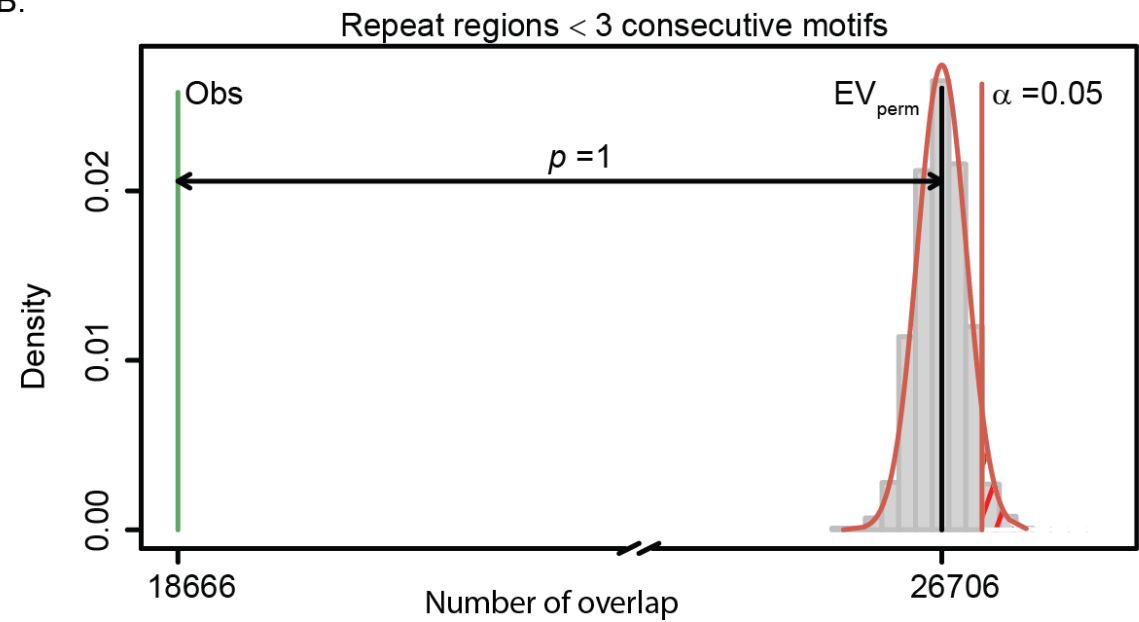

C.

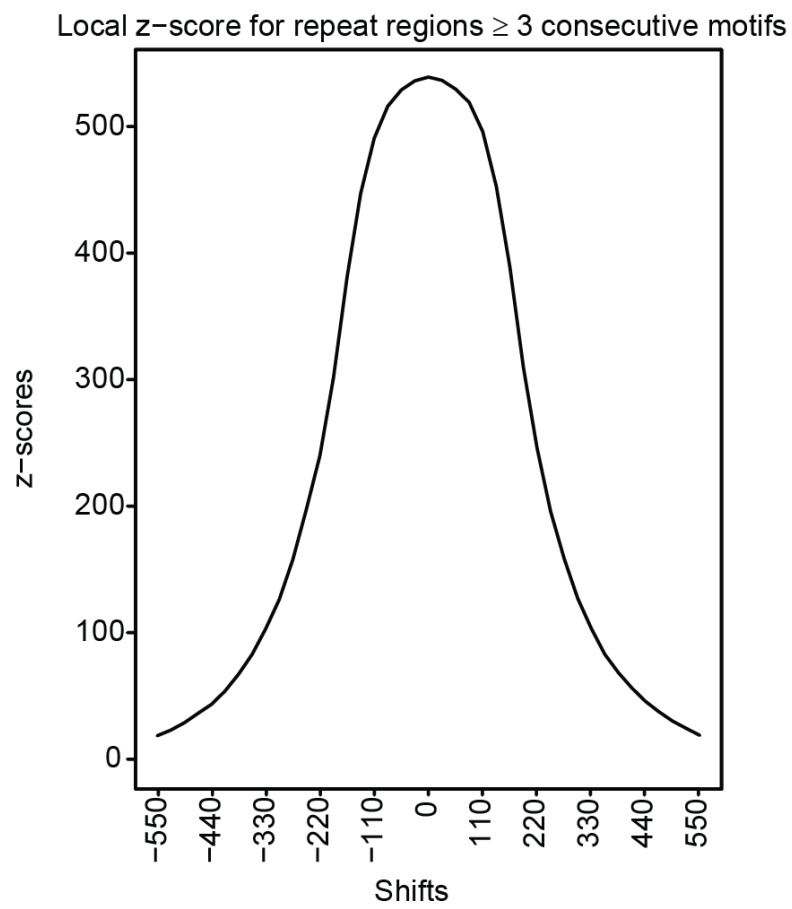

D.

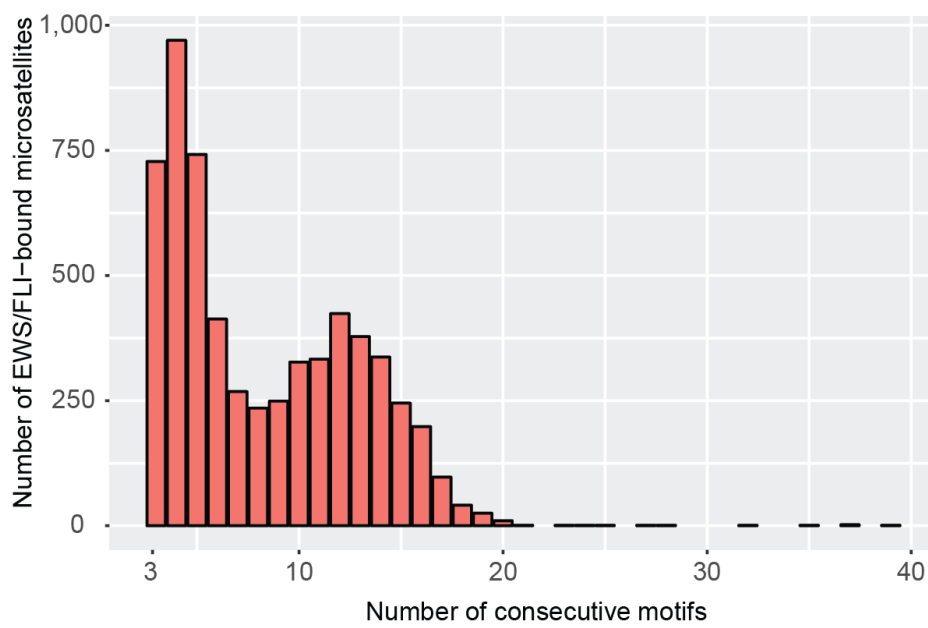

Supplement: S1 Fig — (A) Histogram of number of EWS/FLI peaks per Mb (normalized by chromosome length) in each chromosome. (B) Permutation test shows that the number of EWS/FLI binding sites that overlap with repeat regions with 2 or less consecutive motifs is not significantly higher than random chance (p = 1). The red line denotes the significance limit (α = 0.05). Gray bars represent the number of overlaps of the random regions with EWS/FLI binding sites. The black line represents the mean and in green the number of overlaps of repeat regions with 2 or less consecutive motifs. EVperm is the expected value of the permutation (number of overlaps in random samples). Obs is the observed number of overlap. (C) Plot of shifted z-score for the association between EWS/FLI repeat regions ≥ 3 consecutive motifs and EWS/FLI binding sites showing that this association is highly dependent on the location of the regions. (D) Number of EWS/FLI-bound microsatellites and the number of consecutive motifs in these microsatellites. (PDF) [file pone.0186275.s001.pdf]

S2 Fig

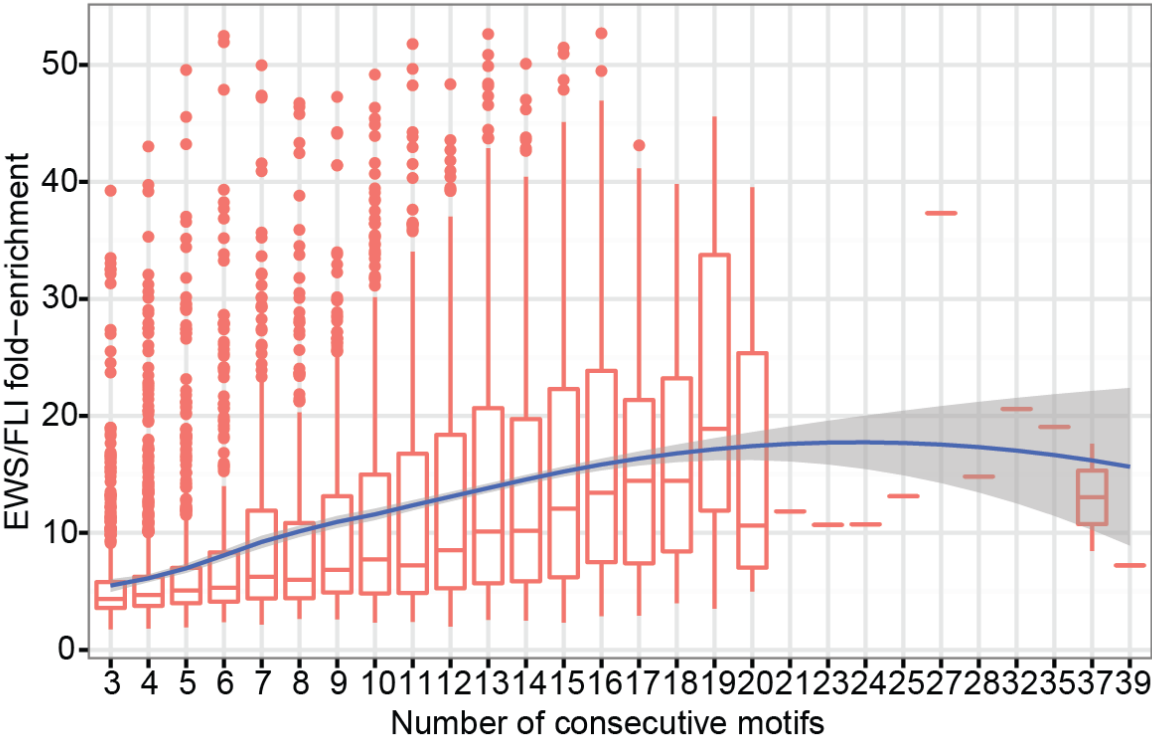

Supplement: S2 Fig — The blue line is the estimated LOESS regression line of the mean with the estimated 95% confidence bands (shaded region). (PDF) [file pone.0186275.s002.pdf]

S3 Fig

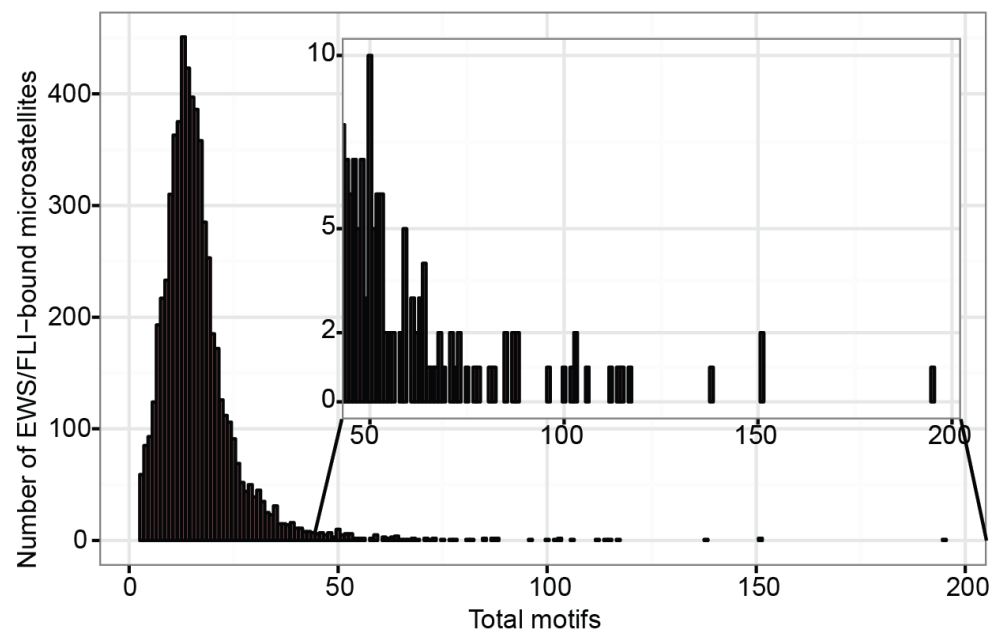

Supplement: S3 Fig — (PDF) [file pone.0186275.s003.pdf]

S4 Fig

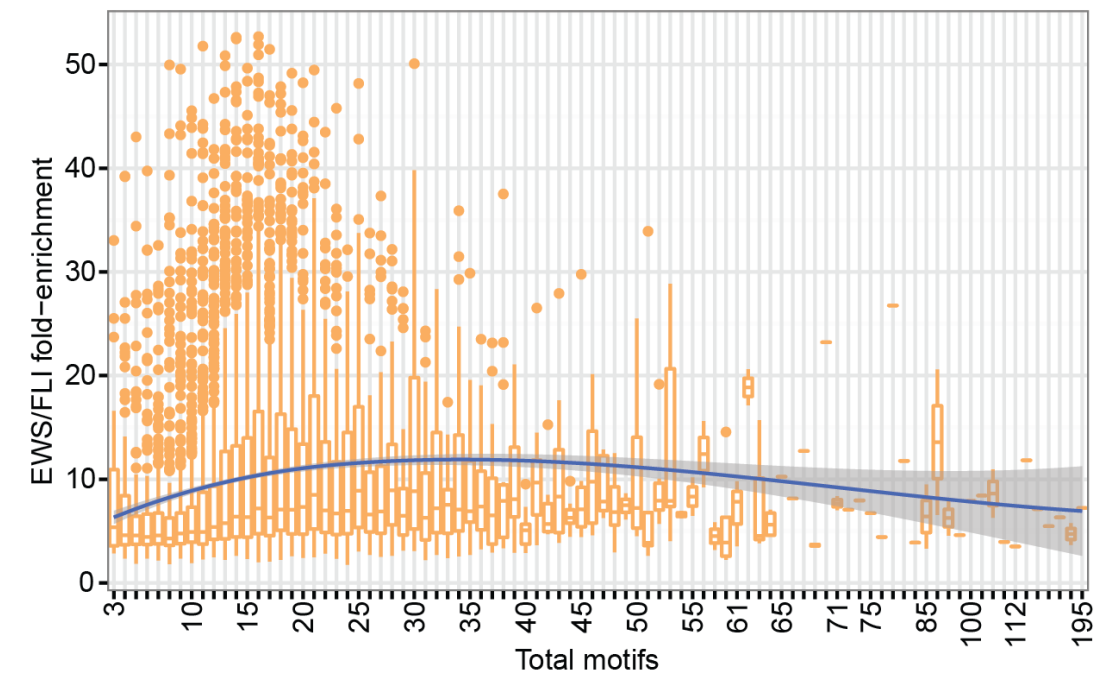

Supplement: S4 Fig — The blue line is the estimated LOESS regression line of the mean with the estimated 95% confidence bands (shaded region). (PDF) [file pone.0186275.s004.pdf]

S5 Fig

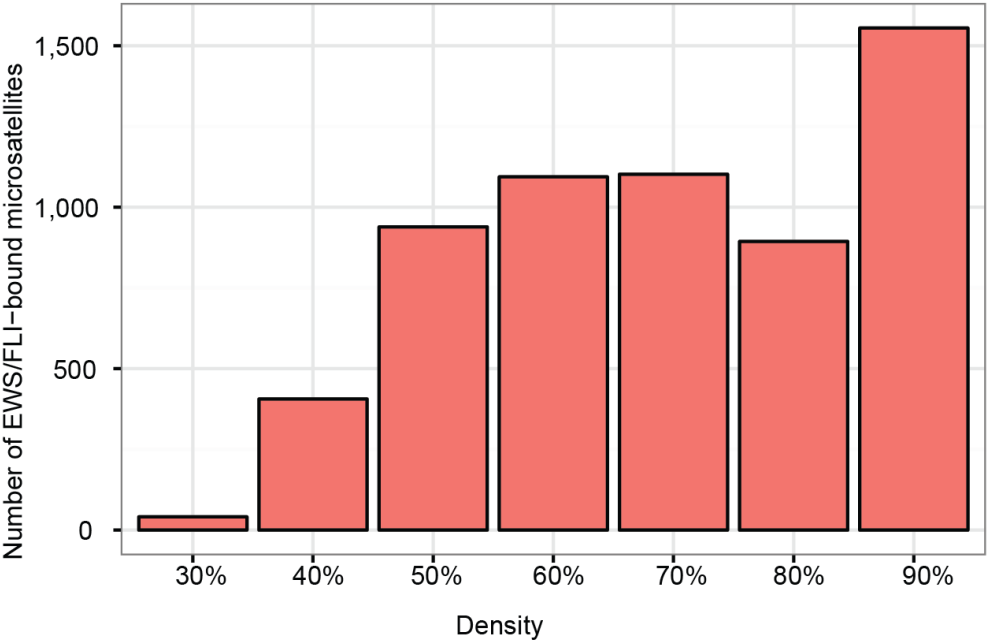

Supplement: S5 Fig — (PDF) [file pone.0186275.s005.pdf]

S6 Fig

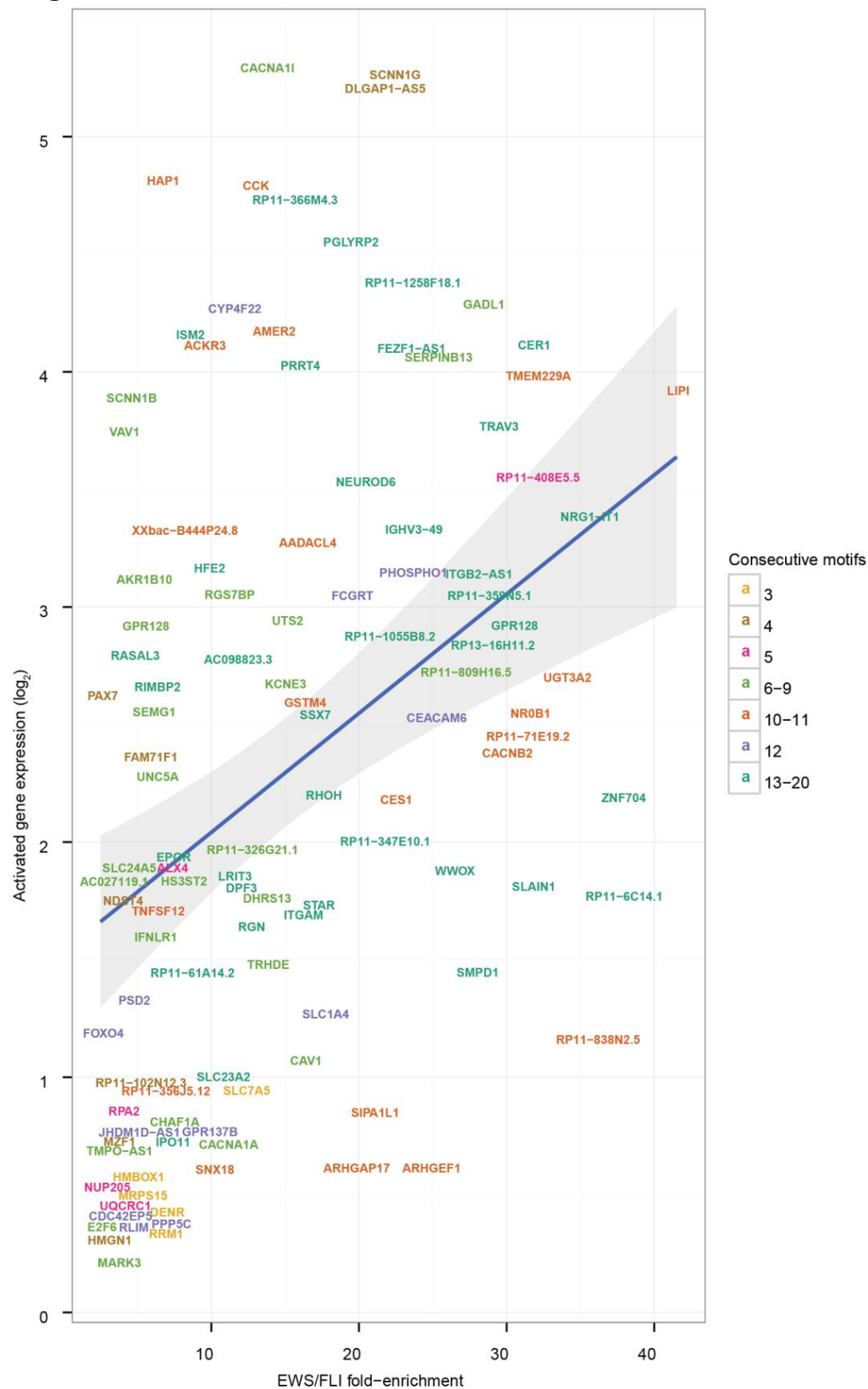

Supplement: S6 Fig — Scatter plot showing activated gene names (False Discovery Rate (FDR) ≤ 5%) that are within 5kb of microsatellites with their EWS/FLI fold-enrichment and their corresponding gene expression (log2). Note: some gene names are adjusted for readability. (PDF) [file pone.0186275.s006.pdf]

**S7 Fig**

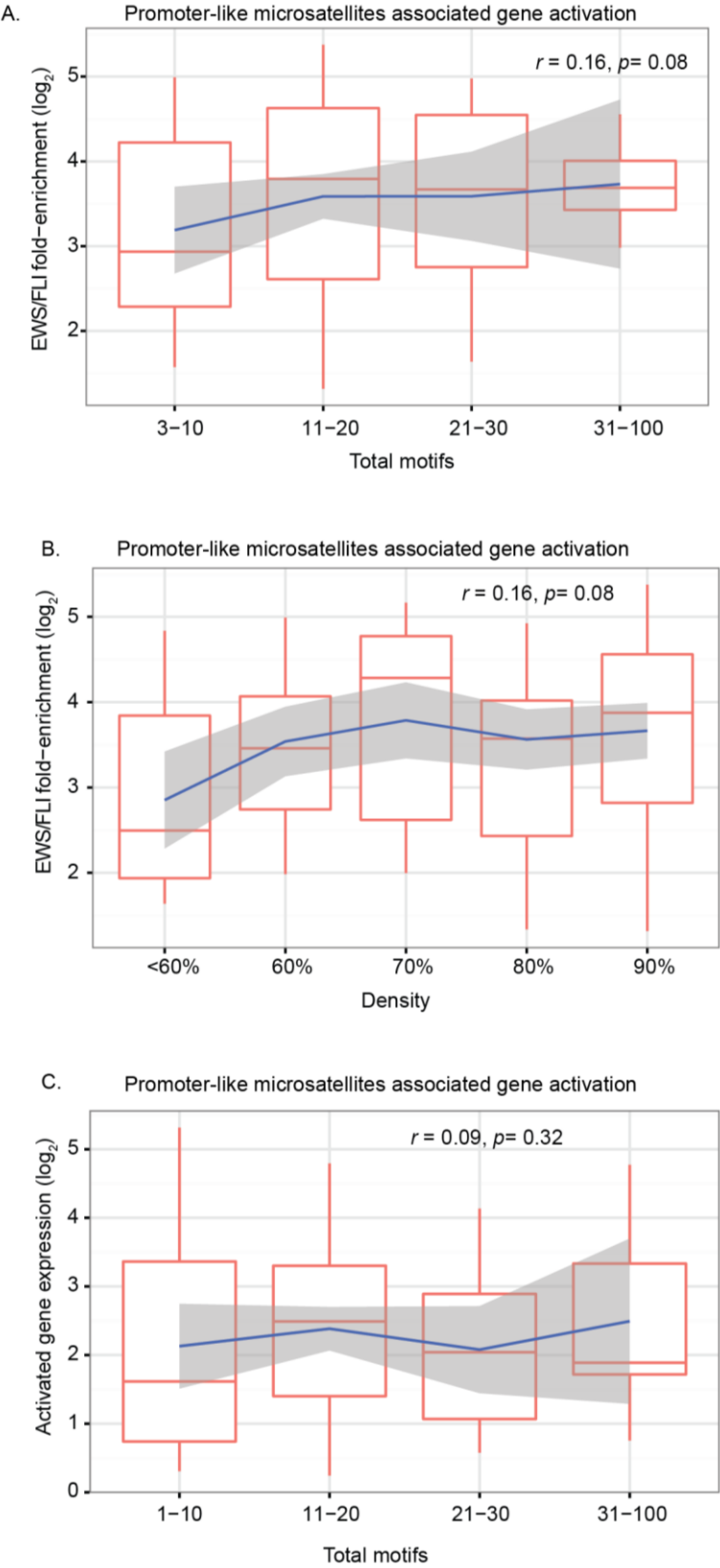

D. Correlation between EWS/FLI binding intensities and consecutive motifs in SKNMC cells

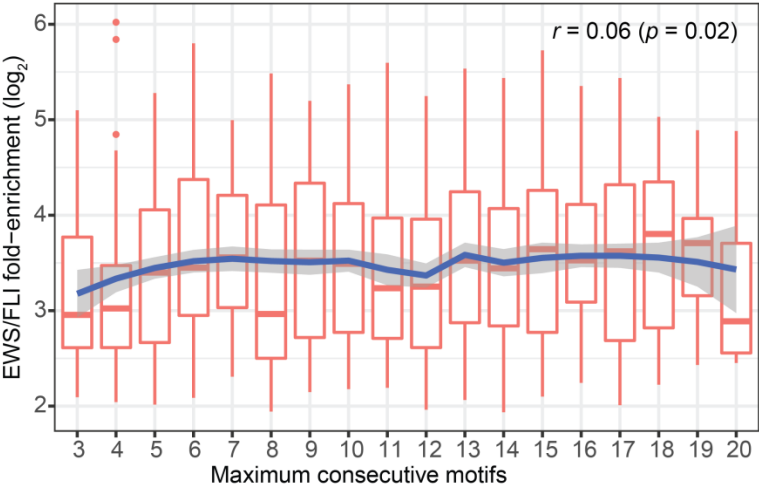

Supplement: S7 Fig — (A) Trend toward positive correlation between total motifs of EWS/FLI-bound microsatellites and EWS/FLI fold-enrichment (log2). (B) Trend toward positive correlation between densities of EWS/FLI-bound microsatellites and EWS/FLI fold-enrichment (log2). (C) No significant correlation between total motifs of EWS/FLI bound microsatellites and activated gene expression (log2). LOESS regression line is shown in blue. Shaded region is the estimated 95% confidence bands.(D) Trend toward positive correlation between EWS/FLI fold-enrichment (log2) and number of consecutive motifs in SK-N-MC cells (r = 0.06, p = 0.02). (PDF) [file pone.0186275.s007.pdf]

**S9 Fig**

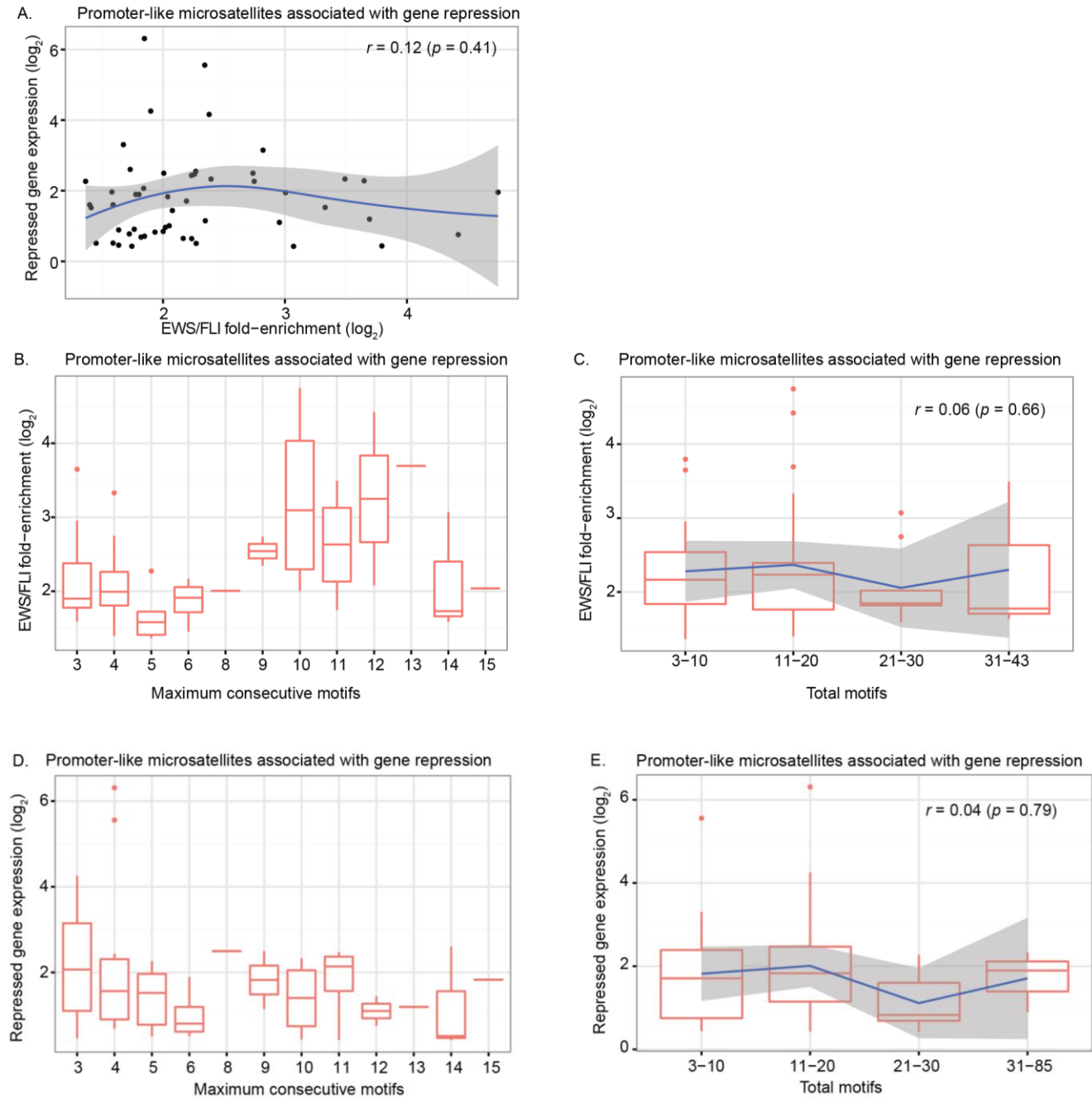

Supplement: S9 Fig — (A) No correlation between EWS/FLI fold-enrichment and gene expression (r = 0.12, p = 0.41). (B) No correlation between EWS/FLI fold-enrichment and number of consecutive motifs (r = 0.18, p = 0.19). (C) No correlation between EWS/FLI fold-enrichment and total motifs (r = 0.06, p = 0.66). (D) No correlation between gene expression and number of consecutive motifs (r = -0.22, p = 0.12). (E) No correlation between gene expression and total number of motifs (r = -0.04, p = 0.79). Shaded region is the 95% confidence interval. (PDF) [file pone.0186275.s009.pdf]

S11 Fig

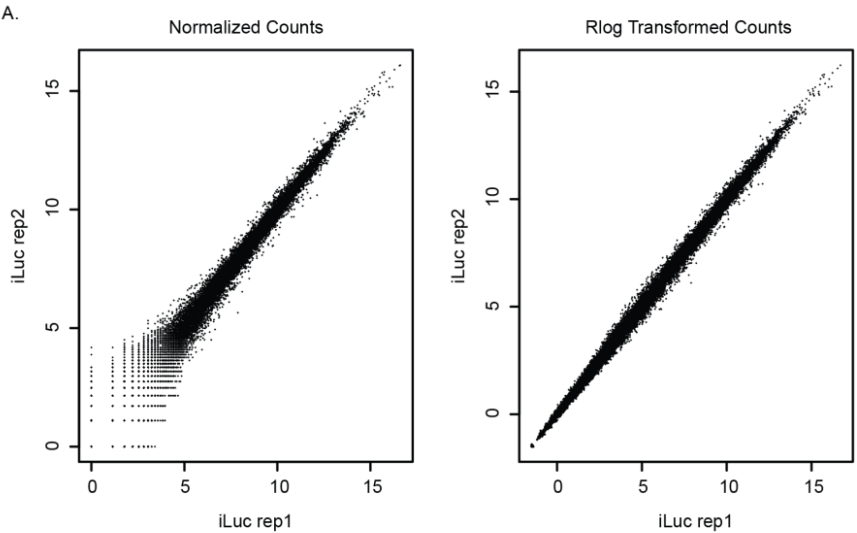

B. Heatmap of sample-to-sample distances using rlog-transformed counts

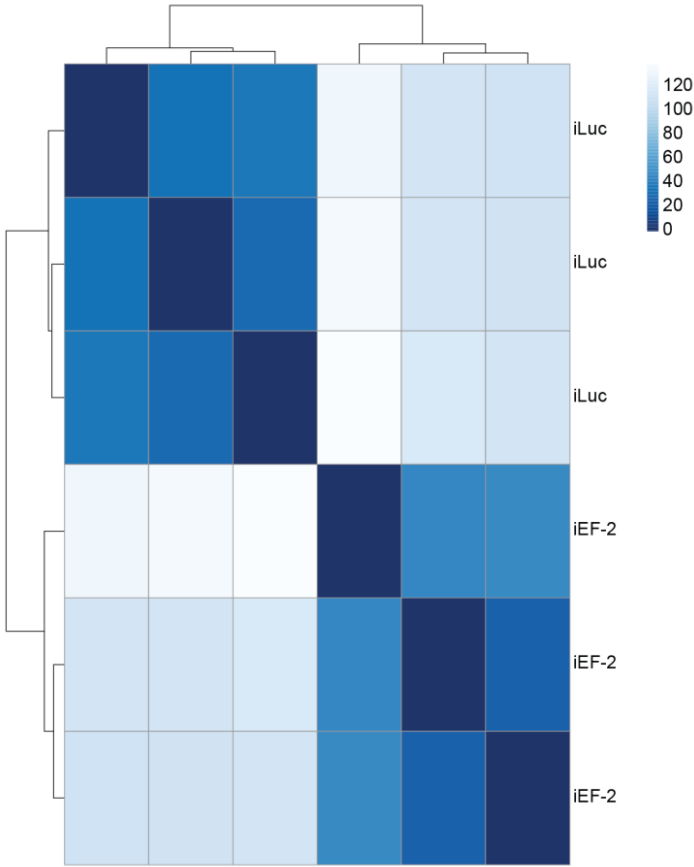

Supplement: S11 Fig — (A) Comparison of two different normalization methods. Left panel, counts normalized by sequencing depth. Right panel, counts normalized by rlog transformation. Sequencing depth normalization still showing bias toward highly expressed genes (i.e. high variance for low expressed genes), while rlog transformation no longer shows such bias (i.e. variances are stabilized across genes). (B) Heat map of sample-to-sample similarities using rlog transformed counts. Color represents distance between samples with dark blue indicating samples with high similarities. (PDF) [file pone.0186275.s011.pdf]
