# Supplementary material for: Identification of two types of GGAA-microsatellites and their roles in EWS/FLI binding and gene regulation in Ewing sarcoma
Source: PLoS One. 2017 Nov 1;12(11):e0186275. doi: 10.1371/journal.pone.0186275 (PMC5665490; doi:10.1371/journal.pone.0186275)
Supplement: S8 Fig — (A) EWS/FLI fold-enrichment has a significant positive correlation with total number of motifs (r = 0.25, p = 1.28 × 10^−9^). (B) Gene expression has significant but minimal positive correlation with total number of motifs (r = 0.10, p = 0.02). (C) Trend toward minimal positive correlation between activated gene expression and number of consecutive motifs (r = 0.07, p = 0.08). (PDF) [file pone.0186275.s008.pdf]

S8 Fig

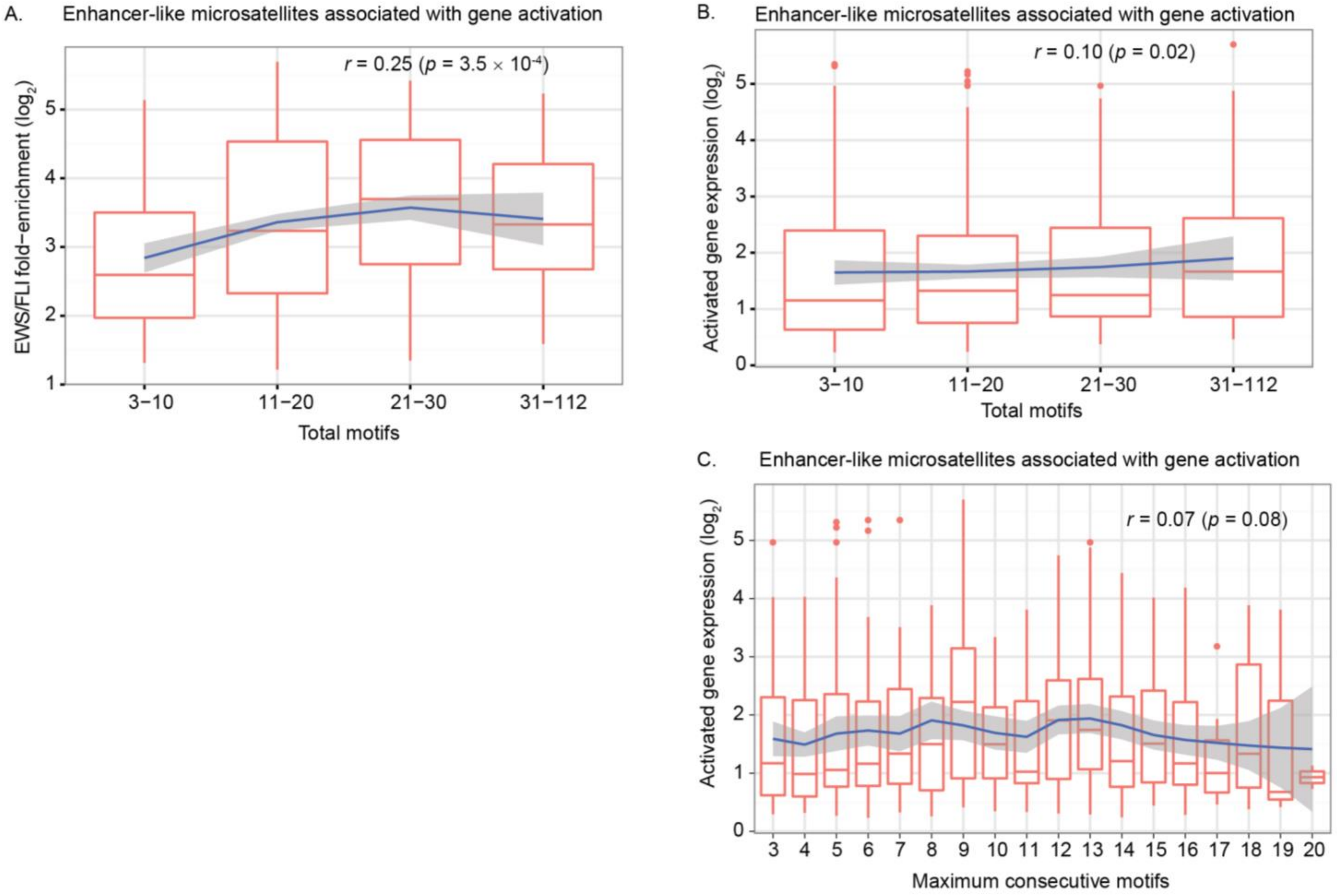
A.
Enhancer-like microsatellites associated with gene activation
r = 0.25 (p = 3.5 × 10⁻⁴)
EWS/FLI fold−enrichment (log2)
1
2
3
4
5
3−10
11−20
21−30
31−112
Total motifs
B.
Enhancer-like microsatellites associated with gene activation
r = 0.10 (p = 0.02)
Activated gene expression (log2)
0
1
2
3
4
5
3−10
11−20
21−30
31−112
Total motifs
C.
Enhancer-like microsatellites associated with gene activation
r = 0.07 (p = 0.08)
Activated gene expression (log2)
0
1
2
3
4
5
3
4
5
6
7
8
9
10
11
12
13
14
15
16
17
18
19
20
Maximum consecutive motifs
