# Supplementary material for: Identification of two types of GGAA-microsatellites and their roles in EWS/FLI binding and gene regulation in Ewing sarcoma
Source: PLoS One. 2017 Nov 1;12(11):e0186275. doi: 10.1371/journal.pone.0186275 (PMC5665490; doi:10.1371/journal.pone.0186275)
Supplement: S10 Fig — (A) Significant positive correlation between EWS/FLI fold-enrichment and number of consecutive motifs (r = 0.40, p < 2.2×10^−4^). (B) No correlation between EWS/FLI fold-enrichment and gene expression (r = -0.05, p = 0.33). (C) No correlation between repressed genes’ expression and number of consecutive motifs (r = -0.04, p = 0.43). LOESS regression line is shown in blue. Shaded region is the estimated 95% confidence bands. (PDF) [file pone.0186275.s010.pdf]

S10 Fig

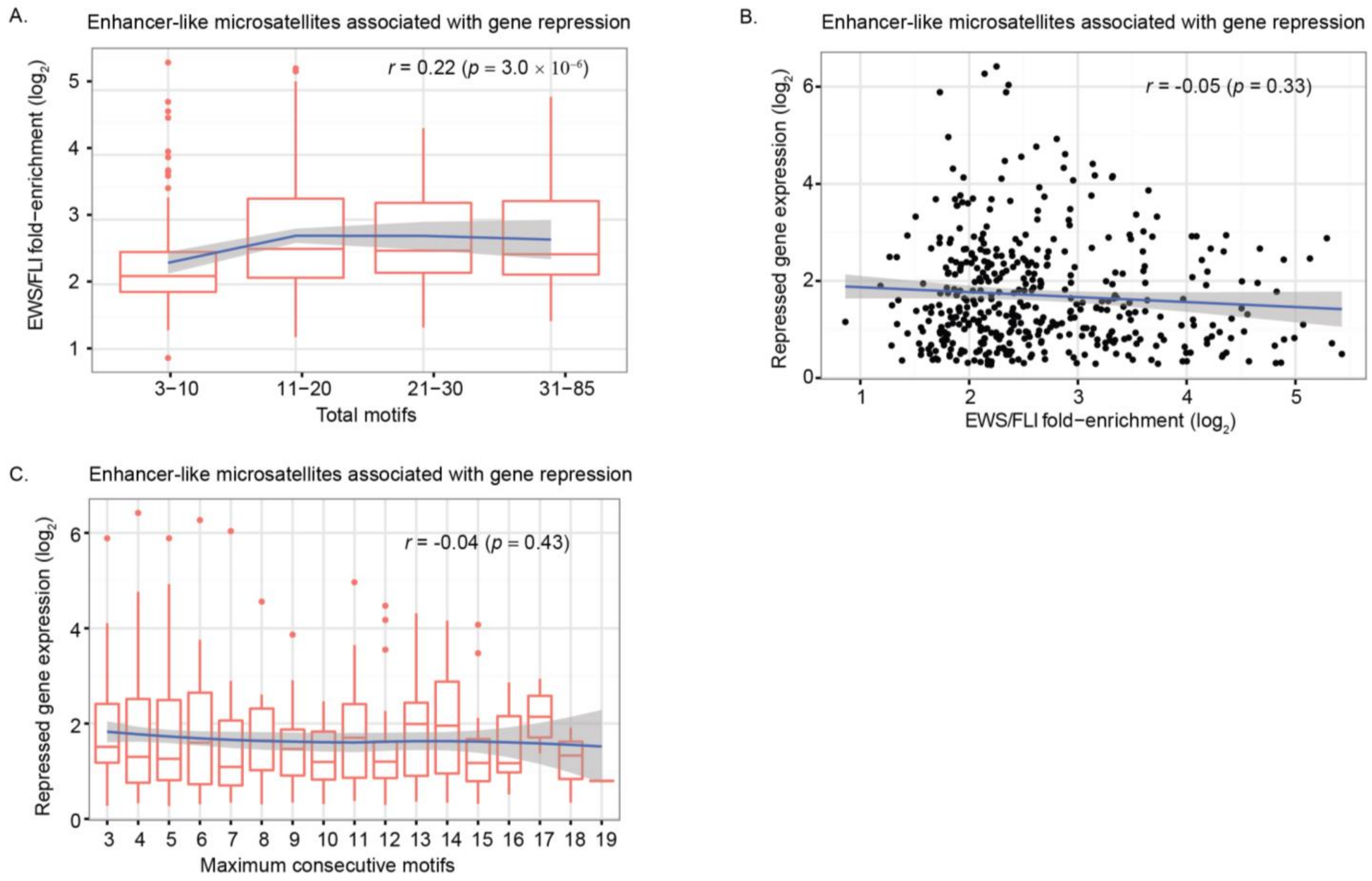
A.
Enhancer-like microsatellites associated with gene repression
r = 0.22 (p = 3.0 × 10−6)
EWS/FLI fold−enrichment (log2)
3−10
11−20
21−30
31−85
Total motifs
B.
Enhancer-like microsatellites associated with gene repression
r = -0.05 (p = 0.33)
Repressed gene expression (log2)
EWS/FLI fold−enrichment (log2)
C.
Enhancer-like microsatellites associated with gene repression
r = -0.04 (p = 0.43)
Repressed gene expression (log2)
Maximum consecutive motifs
