# Supplementary material for: Identification of two types of GGAA-microsatellites and their roles in EWS/FLI binding and gene regulation in Ewing sarcoma
Source: PLoS One. 2017 Nov 1;12(11):e0186275. doi: 10.1371/journal.pone.0186275 (PMC5665490; doi:10.1371/journal.pone.0186275)
Supplement: S1 Table — (PDF) [file pone.0186275.s012.pdf]

S1 Table. Examples of mixed repeat regions (repeat regions that contain both GGAA and TTCC motifs).

	Genomic location (hg19)	Sequence
1.	chr1:199,130,087-199,130,206	**TTCCTTCC**AA**GGAAGGAA**GGGAGGGAGGGA**GGAAGGAAGGAAGGAAGGAA**GAA**GGAAGGAAGGAAGGAAGGAAGGAAGGAA**GAA**GGAAGGAAGGAAGGAAGGAAGGAAGGAAGGAA**
2.	chr4: 34,658,583-34,658,655	**TTCCTTCC**TCA**GGAA**AGGACCTTTGGGCAGCAA**GGAAGGAAGGAAGGAAGGAAGGAA**AGAA**GGAA**GAAA**GGAA**
3.	chr22: 45,138,923 -45,138,773	**GGAA**GGGAGGGAGGGA**GGAAGGAAGGAA**A**GGAAGGAA**GGGAGGGACGAAGGGA**GGAA**GGAGAAAGAAA**GGAA**AGAAA**GGAA**AGATAGAGAA**GGAAGGAA**AGGAGAGA**GGA**AAGGACTCC**TTCCTTCC**TTGCAGTCCCTGTAGCTGCT**TTCC**
4.	chrY: 7,311,313- 7,311,439	**GGAA**ATATGATTCTG**GGAAGGAATTCCTTCCTTCCTTCCTTCCTTCCTTCCTTCCTTCCTTCCTTCC**TTAT**TTCCTTCCTTCCTTCCTTCCTTCC**TTAT**TTCCTTCCTTCCTTCC**TCCCACCT**TTCC**
5.	chr18: 30,686,220-30,686,285	**TTCCTTCCTTCC**TGCAAGT**TTCC**TCCTTTCAATT**TTCC**CAAGAAAATAAAGAAA**GGAAGGAAGGAA**
