# Supplementary material for: Identification of two types of GGAA-microsatellites and their roles in EWS/FLI binding and gene regulation in Ewing sarcoma
Source: PLoS One. 2017 Nov 1;12(11):e0186275. doi: 10.1371/journal.pone.0186275 (PMC5665490; doi:10.1371/journal.pone.0186275)
Supplement: S2 Table — (PDF) [file pone.0186275.s013.pdf]

S2 Table. Correlation between microsatellites and EWS/FLI binding enrichment and EWS/FLI-activated genes.

**Number of microsatellites**	**6,031**
Corr. of peak fold change and number of consecutive motifs, r (p-value)	0.46 (2.2e-16)
Corr. of peak fold change and total number of motifs, r (p-value)	0.23 (2.2e-16)
**Activated genes**	
Number of genes (n)	533
Number of microsatellites (n)	694
Corr. of gene expression and peak fold change, r (p-value)	0.21 (1.6e-08)
Corr. of peak fold change and number of consecutive motif, r (p-value)	0.52 (2.2e-16)
Corr. of peak fold change and total number of motif, r (p-value)	0.24 (1.9e-10)
Corr. of gene expression and number of consecutive motif, r (p-value)	0.12 (1.5e-03)
Corr. of gene expression and total number of motif, r (p-value)	0.11 (5.0e-03)
**Promoter-like microsatellites**	
Number of genes (n)	113
Number of microsatellites (n)	114
Corr. of gene expression and peak fold change, r (p-value)	0.46 (3.3e-07)
Corr. of peak fold change and number of consecutive motif, r (p-value)	0.43 (1.5e-06)
Corr. of peak fold change and total number of motif, r (p-value)	0.16 (0.08)
Corr. of gene expression and number of consecutive motif, r (p-value)	0.23 (0.01)
Corr. of gene expression and total number of motif, r (p-value)	0.09 (0.32)
**Enhancer-like microsatellites**	
Number of genes (n)	440
Number of microsatellites (n)	580
Corr. of gene expression and peak fold change, r (p-value)	0.15 (3.5e-04)
Corr. of peak fold change and number of consecutive motif, r (p-value)	0.53 (2.2e-16)
Corr. of peak fold change and total number of motif, r (p-value)	0.25 (1.28e-09)
Corr. of gene expression and number of consecutive motif, r (p-value)	0.07 (0.08)
Corr. of gene expression and total number of motif, r (p-value)	0.10 (0.02)
