# Supplementary material for: Identification of two types of GGAA-microsatellites and their roles in EWS/FLI binding and gene regulation in Ewing sarcoma
Source: PLoS One. 2017 Nov 1;12(11):e0186275. doi: 10.1371/journal.pone.0186275 (PMC5665490; doi:10.1371/journal.pone.0186275)
Supplement: S3 Table — (PDF) [file pone.0186275.s014.pdf]

S3 Table. Correlation between microsatellites, EWS/FLI binding enrichment and EWS/FLI-repressed genes.

**Number of microsatellites**	**6031**
Corr. of peak fold change and number of consecutive motifs, r (p-value)	0.46 (2.2e-16)
Corr. of peak fold change and total number of motifs, r (p-value)	0.23 (2.2e-16)
**Repressed genes**	
Number of genes	400
Number of microsatellites	477
Corr. of gene expression and peak fold change r (p-value)	-0.03 (0.51)
Corr. of peak fold change and number of consecutive motif, r (p-value)	0.39 (2.2e-16)
Corr. of peak fold change and total number of motif, r (p-value)	0.21 (3.1e-06)
Corr. of gene expression and number of consecutive motif r (p-value)	-0.06 (0.21)
Corr. of gene expression and number of total motif r (p-value)	-0.02 (0.62)
**Promoter-like microsatellites**	
Number of genes	49
Number of microsatellites	52
Corr. of gene expression and peak fold change, r (p-value)	0.12 (0.41)
Corr. of peak fold change and number of consecutive motif, r (p-value)	0.18 (0.19)
Corr. of peak fold change and total number of motif, r (p-value)	0.06 (0.66)
Corr. of gene expression and number of consecutive motif, r (p-value)	-0.22 (0.12)
Corr. of gene expression and total number of motif, r (p-value)	-0.04 (0.79)
**Enhancer-like microsatellites**	
Number of genes	357
Number of microsatellites	425
Corr. of gene expression and peak fold change, r (p-value)	-0.05 (0.33)
Corr. of peak fold change and number of consecutive motif, r (p-value)	0.40 (2.2e-16)
Corr. of peak fold change and total number of motif, r (p-value)	0.22 (3.0e-6)
Corr. of gene expression and number of consecutive motif, r (p-value)	-0.04 (0.43)
Corr. of gene expression and total number of motif, r (p-value)	-0.02 (0.68)
